# Diazabicyclo analogues of maraviroc: synthesis, modeling, NMR studies and antiviral activity†
†The authors declare no competing interests.
‡
‡Electronic supplementary information (ESI) available. See DOI: 10.1039/c6md00575f


**DOI:** 10.1039/c6md00575f

**Published:** 2016-12-16

**Authors:** L. Legnani, D. Colombo, A. Venuti, C. Pastori, L. Lopalco, L. Toma, M. Mori, G. Grazioso, S. Villa

**Affiliations:** a Dipartimento di Chimica , Università di Pavia , Via Taramelli 12 , 27100 Pavia , Italy; b Dipartimento di Scienze del Farmaco , Università di Catania , V.le A. Doria 6 , 95125 Catania , Italy; c Dipartimento di Biotecnologie Mediche e Medicina Traslazionale , Università di Milano , Via Saldini 50 , 20133 Milano , Italy; d Division of Immunology, Transplantation and Infectious Diseases , San Raffaele Scientific Institute , Milan , Italy; e Dipartimento di Scienze Farmaceutiche , Università di Milano , Via L. Mangiagalli 25 , 20133 Milano , Italy . Email: stefania.villa@unimi.it ; Fax: +39 02 503 19359 ; Tel: +39 02 503 19368

## Abstract

Two diazabicyclo analogues of maraviroc, in which the azabicyclooctane moiety is replaced by diazabicyclooctane or diazabicyclononane, were synthesized and tested, through a viral neutralization assay, on a panel of six pseudoviruses. The diazabicyclooctane derivative maintained a significant infectivity reduction power, whereas the diazabicyclononane was less effective. Biological data were rationalized through a computational study that allowed the conformational preferences of the compounds to be determined and a correlation between the inhibitory activity, the bridge length of the bicycle, and the rotational barrier around dihedral angle *τ*_7_ to be hypothesized. A high-field NMR analysis supported the modeling results.

## Abstract 2

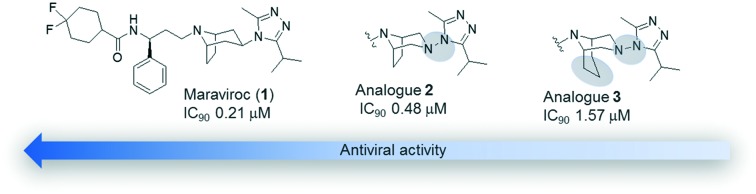
Two diazabicyclo analogues of maraviroc, in which the azabicyclooctane moiety is replaced by diazabicyclooctane or diazabicyclononane, were synthesized and tested, through a viral neutralization assay, on a panel of six pseudoviruses.

## Introduction

AIDS, a disease caused by the Human Immunodeficiency Virus (HIV), is pandemic at the global level^1^ and, in the absence of an effective preventive vaccine, all world health organizations consider anti-HIV drugs a priority. A growing research field in drug discovery is focused on the identification of new entry inhibitors^2^ capable of interfering with the events that occur between the anchorage of the virion to CD4 and the fusion of the two membranes.

It is known that the HIV-1 entry process in a host cell depends on the interaction of the viral envelope protein gp120 with the receptor/coreceptors located on the host cell surface. Because CCR5 is the predominant co-receptor for clinical HIV isolates, it has become a very attractive target for anti-HIV therapy. All CCR5 antagonists inhibit HIV-1 entry into target cells by blocking the interaction between gp120 and CCR5.

Nowadays, this still seems one of the most promising therapeutic approaches to treat HIV-1 infection in support of the Highly Active Anti-Retroviral Therapy (HAART).^3^

**Fig. 1:**
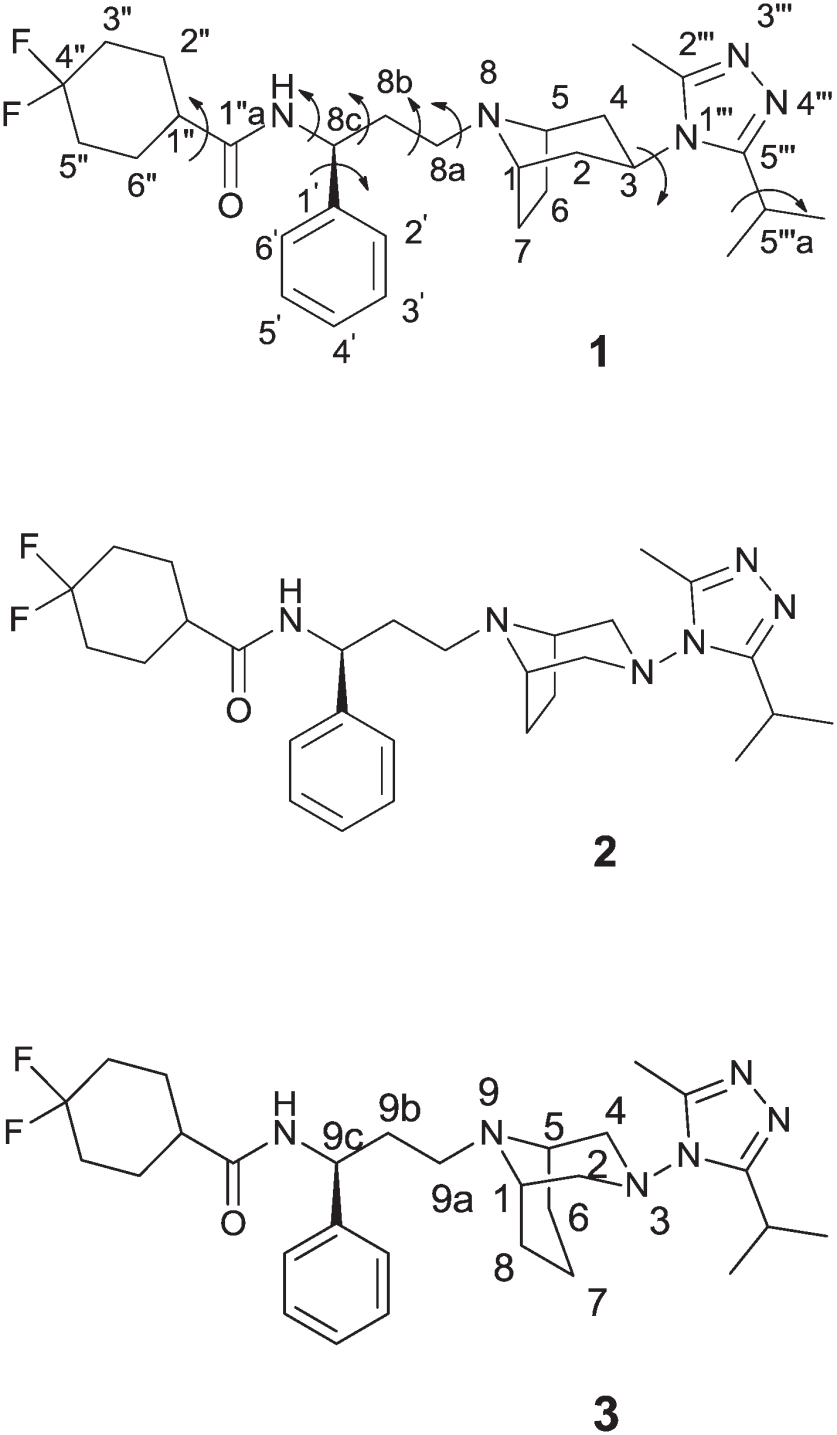
Structures of maraviroc (**1**) and its diazabicyclo analogues **2** and **3**.

Maraviroc (**1**, Fig. 1), a triazolotropane-based compound synthesized by Pfizer,^4^ is the only CCR5 inhibitor that has been approved by both the US FDA and the European Medicines Agency (EMA) for the treatment of antiretroviral drug-experienced and naive patients.^5^ It is sold under the trade name Selzentry (Celsentri outside the United States) as film-coated tablets for oral administration.^6^ It shows good antiviral potency and pharmacological properties,^7^ being active against 200 clinically derived HIV-1 envelope-recombinant pseudoviruses, 100 of which are obtained from viruses resistant to existing drug classes.^8^ Nevertheless, FDA raised concerns that it could be associated with an increased risk of liver damage, lymphoma, infections and heart attack.^9^

Recently, the crystal structure of human CCR5 bound to **1** has been reported^10^ revealing several details of the allosteric inhibition mechanism played by the ligand. In fact, at variance with other chemokines involved in the viral entry, the binding site of maraviroc is positioned in a region shaped by helices I, II, III, V, VI and VII. In this site, the nitrogen atom of tropane is protonated and engaged in a salt-bridge interaction with Glu-283 and the carboxamide nitrogen participates in a hydrogen bond with Tyr-251; the length of the carbon chain between these two nitrogen atoms is considered critical for the anti-HIV infection activity. Additionally, nitrogen atoms N3 and N4 (Fig. 1) of the triazole ring form hydrogen bonds with Tyr-37 and a water molecule located in close proximity to the ligand, respectively. A fluorine atom takes part in two hydrogen bonds with Thr-195 and Thr-259. The phenyl group, the triazole, and the cyclohexane moieties make hydrophobic interactions with the surrounding CCR5 residues, whereas the tropane ring protrudes in a region occupied by a solvent molecule (W1220), stabilized by two hydrogen bonds with the side chains of Thr105 and Cys178.

Starting from **1**, the design of structural analogues represents an important tool for the attainment of new compounds endowed with higher potency and devoid of the above described side effects. Thus, in analogy with other small molecules able to bind CCR5 that show a diazotated ring (such as vicriviroc *vs.* SCH-C),^11^ and considering our long-lasting experience in the synthesis and modeling of pharmacologically active compounds^12^ with a diazabicyclo moiety,^13^ we planned to introduce a second nitrogen atom in the bicyclic structure of maraviroc (compound **2**, Fig. 1). A compound having the same structure was actually present in a patent, but without synthetic evidence and biological evaluation.^14^

Moreover, on the basis of the outcomes derived from the analyses of the crystal structure, a ligand able to displace the above-mentioned water molecule (W1220) could be theoretically more active than the template structure, for an entropic gain. Thus, the introduction of an additional methylene group into the bicyclic ring could provide this effect (compound **3**, Fig. 1). In fact, a similar approach has been successfully adopted to improve the binding affinity or selectivity profile of a group of HIV-1 protease inhibitors^15^ and nicotinic ligands.^16^

Therefore, analogues of **1**, in which the tropane moiety is replaced by the 3,8-diazabicyclo[3.2.1]octane and 3,9-diazabicyclo[3.3.1]nonane systems (**2** and **3**, respectively) (Fig. 1), were synthesized and submitted to a viral neutralization assay^17^ in order to investigate their HIV-1 inhibitory activity in comparison with **1**. To explain the obtained results, conformational analysis, NMR analysis and docking studies were also performed.

## Results and discussion

### General procedures for the synthesis of maraviroc analogues

**Scheme 1:**
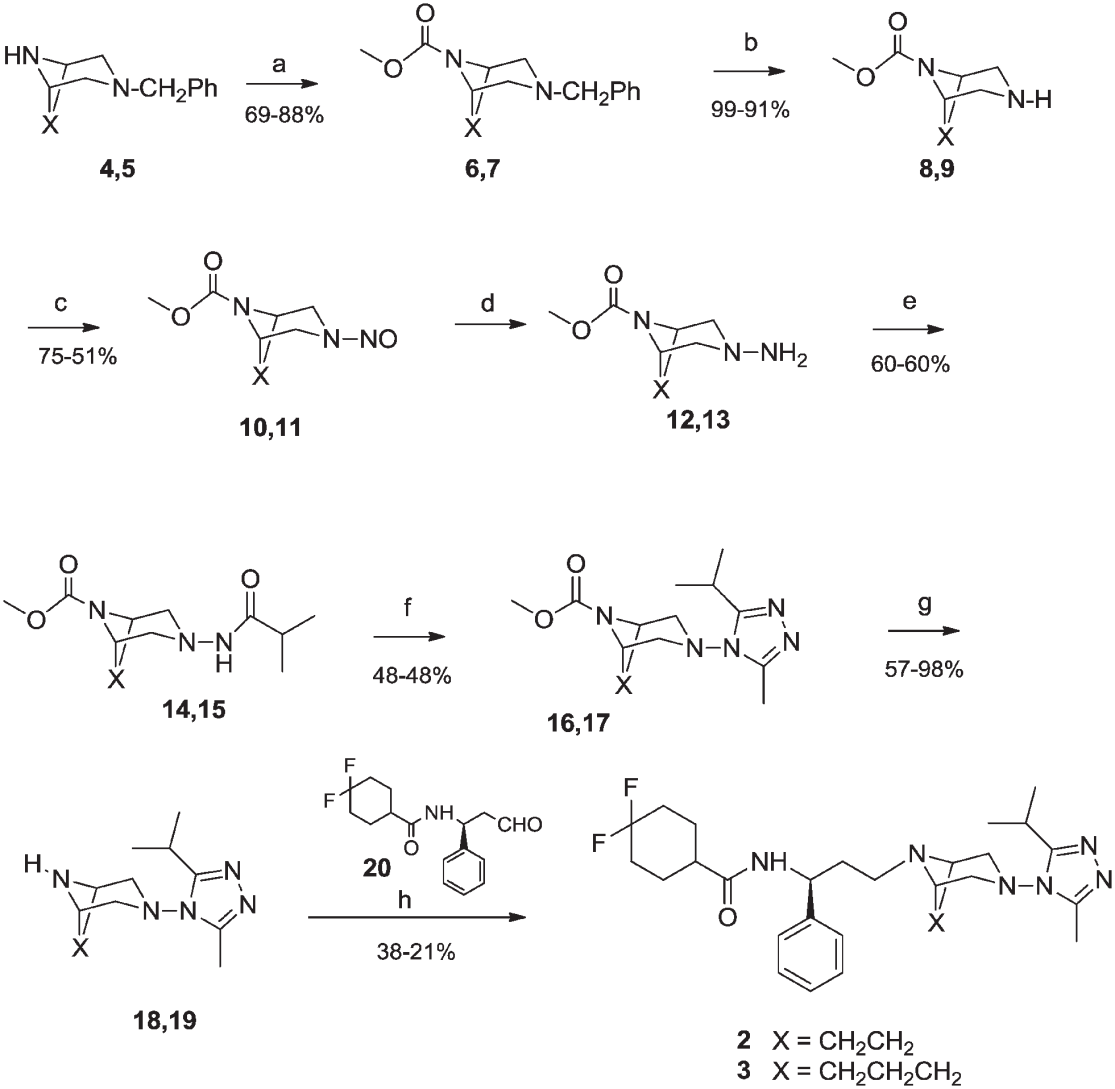
Reagents and conditions: (a) ClCOOCH_3_, TEA, Et_2_O, 0 °C–r.t., 20 h. (b) H_2_, Pd/C 10%, EtOH. (c) aq NaNO_2_, HCl 2 N, 0 °C, N_2_ atm. (d) Zn powder, AcOH, H_2_O, N_2_ atm. (e) Isobutyric acid, TEA, HATU, DMF, 17 h. (f) 1) PCl_5_, CH_2_Cl_2_, 0 °C; 2) AcNHNH_2_, 2-methyl-2-butanol; 3) AcOH, 2-methyl-2-butanol, 80 °C. (g) aq NaOH 10%, MeOH, 80 °C; (h) NaBH(OAc)_3_, CH_2_Cl_2_, AcOH.

Maraviroc derivatives **2** and **3** were synthesized (Scheme 1) with a procedure similar to that already used for the reference compound **1**,^18^ starting from known diazabicyclo precursors **4** and **5**.^19^ After the proper protection of nitrogen at position 8 or 9 in the diazabicyclo scaffold with methyl chloroformate (**6**, **7**) and debenzylation steps by catalytic hydrogenation, using a heterogeneous Pd/C catalyst, **4** and **5** were converted into the intermediates **8** and **9**. Their treatment with sodium nitrite in hydrochloric acid afforded the nitroso derivatives **10** and **11** that were immediately reduced by reaction with zinc in acetic acid to the corresponding amino derivatives **12** and **13**.^20^ The amino groups were acylated with isobutyric acid, using a condensing agent (HATU) to obtain the acyl derivatives **14** and **15** that were converted into the triazoles **16** and **17** through a three-step reaction: activation to the corresponding imidoyl chloride by PCl_5_, trapping with acetic hydrazide, and finally acid-catalyzed cyclization to give the triazole.

Furthermore, derivatives **16** and **17** were deprotected by hydrolysis to **18** and **19**. Finally, coupling of aldehyde **20** (ref. 21) with diazabicyclo derivatives **18** and **19***via* reductive amination in the presence of sodium triacetoxyborohydride in dichloromethane afforded the final compounds **2** and **3**.

### Antiviral activity

A standardized neutralization assay^17^ was performed to assess the infectivity reduction of all compounds under examination; in detail, maraviroc (**1**) and its two analogues **2** and **3** were tested. The infectivity reduction power was challenged on a panel of six CCR5-dependent pseudoviruses, including one laboratory strain, SF162, four Clade B isolates, QH0692, 6535, PVO, and AC10, and one Clade C primary virus, ZM214.

**Fig. 2:**
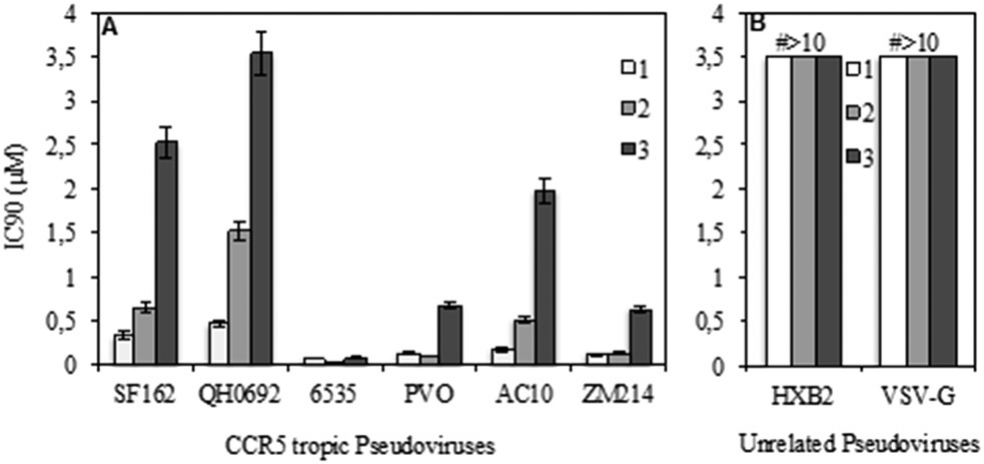
Infectivity reduction of the positive control maraviroc (**1**) and its analogues **2** and **3** on a panel of six CCR5 tropic pseudoviruses (A) and on two unrelated viruses such as HXB2 (CXCR4 tropic) and VSV-G (B). The values are expressed as concentration (μM) leading to 90% of infectivity reduction (IC_90_). Mean plus standard deviations of three independent experiments were shown.

**Fig. 3:**
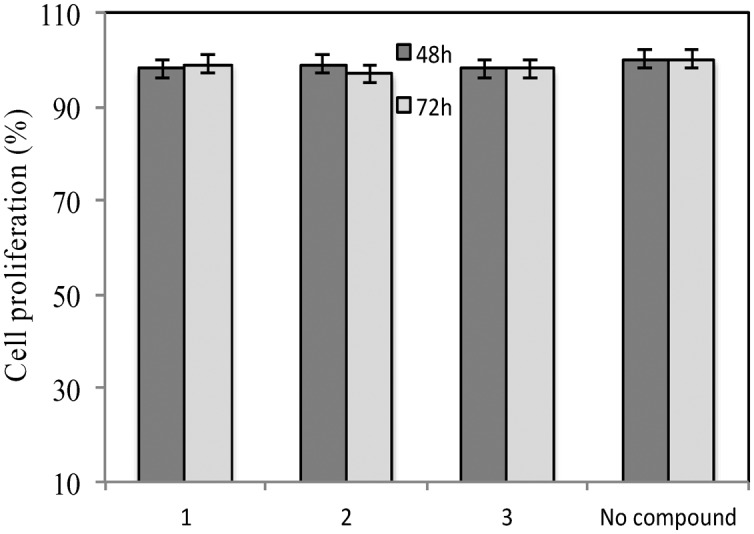
Cell toxicity evaluated at 48 h and 72 h post incubation of the positive control maraviroc (**1**) and its analogues **2** and **3**. The values are expressed as percentage of TZM-bl cell proliferation. 100% represents cells incubated with medium without compounds. Mean plus standard deviations of two independent experiments were shown.

Fig. 2A shows the IC_90_ values (90% of infectivity reduction expressed as concentrations (μM)) obtained by the three compounds as a result of their screening on a panel of six pseudoviruses. As expected, maraviroc (**1**) efficiently neutralized all the tested viruses with a mean IC_90_ of 0.21 μM (ranging from 0.08 to 0.47); compound **2** showed a mean IC_90_ of 0.48 μM (ranging from 0.02 to 1.52). Compound **3** had a lower infectivity reduction power, with a mean IC_90_ of 1.57 μM (ranging from 0.09 to 3.54), thus exhibiting a statistically significant difference from compound **1** (*p* ≤ 0.05). The data showed a progressive decrease of infectivity reduction from **1** to **3**. As negative controls, VSV-G (an HIV-unrelated virus) and HXB2 (CXCR4 tropic virus) were also included in the experiments. All compounds did not reach inhibition of viral infectivity (IC_90_) at the highest concentration (10 μM) with both unrelated viruses, as shown in Fig. 2B. To evaluate the toxicity of each compound to host cells, a longer term viral infectivity test was performed for both inhibition of viral infection and cell proliferation. As the neutralization assay has been standardized and validated,^17^,^22^ for 48 h and 72 h incubation times, we used both incubation times of the cells with each compound. At 72 h of viral infection, the results were very similar to those obtained at 48 h (data not shown). All three compounds did not affect cell proliferation when tested at 10 μM, as shown in Fig. 3.

### Conformational analysis of maraviroc (**1**) and its analogues **2** and **3**

Theoretical calculations were performed within the DFT approach at the B3LYP level with the 6-31G(d) basis set.^23^ Solvent effects were also considered through single-point calculations on the gas-phase optimized geometries, by using a self-consistent reaction field (SCRF) method, based on the polarizable continuum model (PCM),^24^ and water as the solvent. Data provided by the EMA show that in **1** the nitrogen atom of tropane and those of the triazole ring present different p*K*_a_ values, precisely 7.9 and 3.3, respectively, and at physiological pH, only the first one is protonated.^25^ Moreover, docking studies on **1**, reported in the literature, revealed that the positively charged tertiary nitrogen of the tropane moiety could have strong electrostatic interactions in the binding site.^9^,^26^ Thus, particular attention has been paid to the ionizable nitrogen atoms present in the molecules. All the optimizations have been performed on the compounds in their cationic form. Maraviroc (**1**) is a highly flexible molecule with several degrees of conformational freedom related to the orientation around the single bonds exemplified by curved arrows in Fig. 1.

A systematic search of the conformational space of **1** was performed. Firstly, a starting geometry was constructed and optimized. Then, the energy profiles for rotation around the single bonds defined by *τ*_1_–*τ*_8_ were determined with a step size of 30° and the preferences of the difluorocyclohexane and piperidine rings were considered. All the combinations of the observed minima were used to generate the possible starting geometries optimized as above. Tenths of conformations were located and a number of them were populated. Only conformers with a percentage contribution Pi > 1% were considered.^27^

**Fig. 4:**
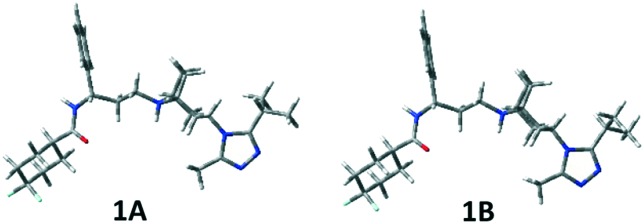
3D-plots of significantly populated conformers of compound **1**.

In Table S1 (see the ESI‡), the relative energies, the equilibrium percentages at 298 K calculated through the Boltzmann equation, and the geometrical features of conformers with Pi > 1% are reported and the corresponding three-dimensional plots are shown in Fig. 4 and S1 (ESI‡). The two preferred conformations **1A** and **1B** are almost identical, a slightly different orientation of the isopropyl group being the only difference. The left portion of the molecule extends equatorially from the tropane nitrogen atom, with the phenyl ring facing the ethylenic tropane bridge, and ends with the carboxyamido function equatorially linked to the difluorocyclohexane ring. The triazole ring is perpendicular to tropane. Conformation **1C** corresponds to **1A** and **1B** with a 180° rotation around the C3–N1′′′ bond.

The two possible orientations described by *τ*_7_, which can be ≊120° or 60°, lead, respectively, to the isopropyl group on the same (**A**, **B**) or on the opposite (**C**, **D**) side of the tropane ethylenic bridge. In **1D–1F**, the three-carbon C8a–C8c chain is folded (as highlighted by the *τ*_4_ values), with the carbonyl oxygen pointing towards H5 at a short distance (about 2 Å). Also, conformations with the carboxamide moiety bonded in the axial position of the difluorocyclohexane ring have been found to be populated (**1G** and **1H**), though with a small contribution to the overall population (∼4%).

**Fig. 5:**
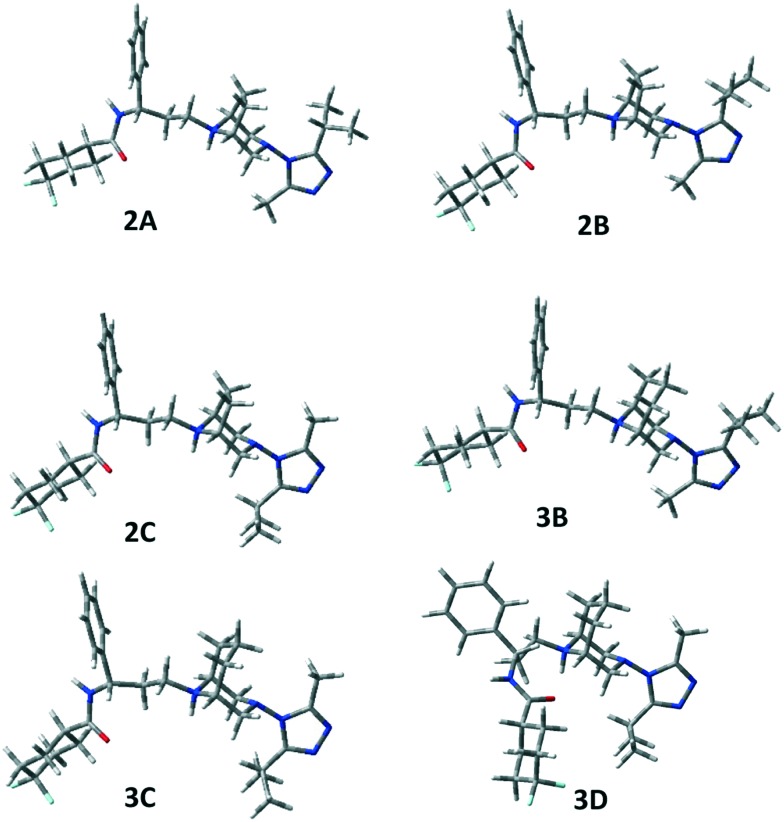
3D-plots of conformers **2A**–**C** and **3B**–**D**.

Then, the same computational approach was applied to compounds **2** and **3**, also considering in these cases their cationic form. We assumed that, at physiological pH, protonation occurs only at N8 (compound **2**) and N9 (compound **3**) in spite of the presence of a second nitrogen atom (N3) on the bicyclic system, as N3 is involved in a N–N bond with triazole and the other triazole nitrogen atoms are expected to be much less basic than N8 (N9) in analogy with the reference compound **1** (see the Experimental section). Also for **2** and **3**, tenths of conformations were located, but only some resulted to be significantly populated (Pi > 1%, see Table S2 in the ESI‡ and Fig. 5). In the case of **3**, the number of located conformers was larger, because of the greater conformational freedom of the bicyclic moiety, which gives rise to “boat” geometries that are not significantly populated and hence not reported in Table S2.‡

The same **A**–**C** low energy conformers already found for **1** were located for **2** and **3** but with a reverse relative energy order. In fact, conformers **A** and **B** are significantly populated (**2A** 21.1%, **3A** 7.0%, **2B** 28.0%, **3B** 11.0%), but the global energy minimum is, in both cases, conformer **C**, showing a different orientation of the triazole ring (see *τ*_7_). However, **2A–C** and **3A–C** represent about 88% and 49% of the overall population, respectively.

Moreover, for **3**, conformer **3D**, showing a different value of *τ*_6_ with respect to **3C**, but the orientation of the isopropyl group on the opposite side of the tropane ethylenic bridge, resulted to be populated (26.1%). In the case of **3**, one populated conformer (**3I**) shows the carboxamide moiety bonded in the axial position of the difluorocyclohexane ring (2.4%).

No significant differences have been observed between the corresponding geometries of the three compounds, but only a different distribution of the population of the various conformations has been observed.

The geometry of **1** in the crystal structure^10^ was very similar to that of **1D**, showing the isopropyl group on the opposite side of the bicyclic system bridge.

On the contrary, the global minimum **1A** showed the isopropyl group on the same side of the bridge.

Therefore, in the last part of the modeling study, we evaluated the interconversion barrier around *τ*_7_ describing the orientation of the isopropyl group. In the case of **1**, the corresponding transition state (TS) had an energy of 12.41 kcal mol^–1^, for **2** of 20.09 kcal mol^–1^ and for **3** of 16.74 kcal mol^–1^. These data indicated that the introduction of the supplementary nitrogen atom (the only structural difference between **1** and **2**) affected the rotational barrier around *τ*_7_ and consequently the position of the isopropyl group with respect to the bridge that could address the antiviral activity. The computed height of the barriers for **2** and **3** is high enough to give rise to distinguishable conformations on the NMR timescale.

### NMR spectroscopy

^1^H NMR chemical shifts of compounds **1–3** were obtained through high field 1D and 2D spectroscopy and the data are shown in Table S3.‡ Starting from the characteristic H-3 (**1**), H-8c (**2**), H-9c (**3**), and H-1′′, H-5′′′a, H-1 and H-5 (**1–3**), easily assigned by HSQC and COSY, the other resonances were assigned. Interestingly, some signals, specifically methyl, isopropyl methyls and H-5′′′a of compounds **2** and **3**, appeared as couples of signals (see Table S3‡), which exhibited coalescence when recorded at increasing temperature (see the ESI‡), suggesting the presence of slowly interconverting conformations as indicated also by their positive cross peaks in the NOESY experiments, due to chemical exchange (see the ESI‡).

NOESY experiments allowed the assignment of the axial and equatorial protons of the bicyclic systems (see the ESI‡).

NOESY data also confirmed the presence of the expected conformers **A**–**F** of **1–3** (Tables S1 and S2‡). Hence, for compound **1**, H-3 (4.41 ppm) cross peaks with H-5′′′a (3.26 ppm) and 2CH_3_-iPr (1.35 ppm) and a cross peak between axial H-2/H-4 (2.29 ppm) and CH_3_ (2.51 ppm) accounted for the triazole isopropyl facing the ethylenic bridge of conformers **1A**, **1B**, **1E** and **1F**, whereas a cross peak of H-3 (4.41 ppm) with CH_3_ at 2.51 ppm accounted for the **1C** and **1D** conformers with the isopropyl placed at the opposite side of the molecule. **1C** and **1D** were also supported by a cross peak between H-5′′′a (3.26 ppm) and H-2ax/H-4ax (2.29 ppm). Finally, a cross peak of H-1/H-5 (3.50/3.53 ppm) with H-8c (5.03 ppm) and with H-8b (2.03 ppm) accounted for **1D–F**.

Analogously, a cross peak between H-5′′′a (3.26 ppm) and H-6/H-7 (2.34 ppm) and between axial H-2/H-4 (4.11 ppm) and CH_3_ (2.70 ppm) accounted for **2A**, **2B**, **2E** and **2F** conformers, whereas a cross peak of H-5′′′a (3.63 ppm) with axial H-2/H-4 (4.11 ppm) and of CH_3_ (2.47 ppm) with equatorial H-2/H-4 (3.35 ppm) accounted for **2C** and **2D**. The H-1/H-5 (4.30/4.21 ppm)–H-8b (2.40 ppm) contact supported the **2D–F** conformations.

The NOESY spectrum of compound **3** showed correlations similar to those found for **1** and **2**. Thus, the **3A**, **3B**, **3E** and **3F** family was confirmed by a cross peak between axial H-2/H-4 (3.99/3.95 ppm) and CH_3_ (2.54 ppm), and by a contact between H-5′′′a (3.15 ppm) and axial H-7 (2.58 ppm). H-5′′′a (3.15 ppm) and axial H-2/H-4 (3.99/3.95 ppm) correlation accounted for **3C** and **3D** and a cross peak between H-1/H-5 (3.59/3.55 ppm) and H-9c (4.99 ppm) was related to **3D–F** conformers. Actually, the observed contacts correspond to distances of less than 3 Å, as measured on the computed most populated conformations of compounds **1–3** (Fig. 4, S1‡ and 5).

### Docking studies

Biological assays suggested that compounds **2** and **3** were slightly less active than the reference one, although minor changes in the structure have been made. This observed decrease of activity could be due to several factors, among them the non-optimal orientation of the ligands in the CCR5 binding site. Therefore, to shed some light on the putative binding mode of **2** and **3**, docking calculations were performed.

The lowest energy conformers of compounds **1–3** were docked into the ligand binding site of CCR5 identified by the presence of maraviroc (**1**) in the X-ray crystal structure of CCR5 deposited by Tan *et al.*10*a* (Protein Data Bank entry ; 4MBS). This model, suitably checked by molecular modeling tools (see the Experimental section for details), was used as the target protein for docking calculations by the GOLD5.2.2 algorithm.^28^,^29^ Moreover, aiming to evaluate the effect of the presence of W1220 in the binding site on the score calculated for the ligands, runs were carried out considering this water molecule as fixed or missing.

**Fig. 6:**
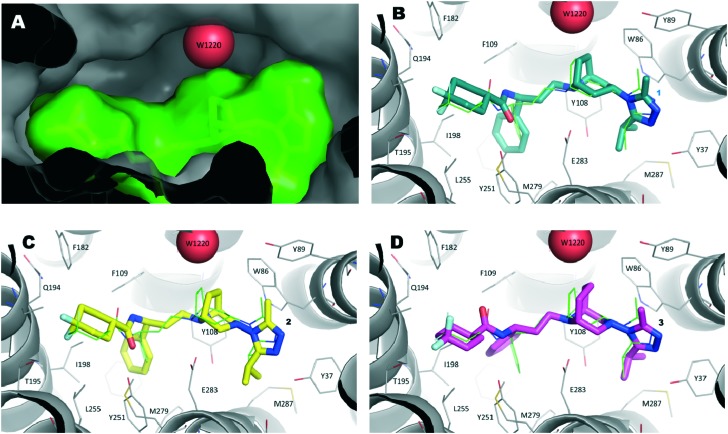
A) Inside view of the ligand binding pocket of maraviroc (**1**) within the CCR5 X-ray crystal structure (PDB code ; 4MBS). Water molecule W1220 is represented as a red sphere and **1** is displayed as a green stick model. The protein and ligand solvent-accessible surface are also shown. B) Binding mode of compound **1** (navy stick model) as predicted by docking calculations. The X-ray pose of **1** is depicted as a thin green stick model. C) Theoretical binding mode of compound **2** (yellow stick model). D) Hypothetical binding mode of compound **3** (magenta stick model).

We initially compared the docking poses obtained for compound **1** with the binding mode evidenced in the X-ray structure (Fig. 6A). As expected, the best scored solution (Fig. 6B, ChemPLP score 123) showed an RMSD value of 1.3 Å with respect to the experimentally determined structure, a good value considering the B factor values (close to 50) of the ligand's atoms in the crystal structure.

Then, by applying the same approach, docking calculations were performed on compound **2**. In this case, the highest scored solution indicated a binding mode strictly resembling that of the template compound (**1**). It was interesting to note that when W1220 was permanently added into the binding site (Fig. 6C), the PLP score was 110 and increased to 115 when W1220 was missing in the binding site. This result was particularly marked when docking calculations were performed on compound **3**. In fact, the best pose of **3** mimicked the X-ray binding mode of **1** and, in the presence of W1220, the ChemPLP score of that pose was 94 (Fig. 6D); whereas, when W1220 was not considered active in the target protein, the ChemPLP score increased to 119, retaining a similar binding mode.

Accordingly, if our compounds were able to displace W1220, compound **3** (ChemPLP score 119) would be as active as **2** (ChemPLP score 115). Conversely, if the water molecules were firmly anchored in the pocket, compound **2** (ChemPLP score 110) should be less potent than **1** (ChemPLP score 123), while compound **3** (ChemPLP score 94) should show lower activity than **2**.

Biological data are in agreement with the second assumption. In fact, maraviroc (**1**) showed an IC_90_ of 0.21 μM, compound **2** showed an IC_90_ of 0.48 μM and compound **3** was three times less active than **2** (IC_90_ = 1.57 μM).

Finally, it is possible to suppose that the simple addition of a small and hydrophobic group (like the methylene moiety) into the tropane ring of compound **1** leads to a detrimental effect on the biological activity of the resulting compound (**3**). Consequently, new maraviroc analogues could be properly designed taking into account that the tropane ring has to be replaced by a bigger and hydrophilic group. This group should be able to displace and mimic the interactions played by W1220, theoretically improving the overall biological activity of the resulting compound.

## Conclusions

Two structural analogues of maraviroc (**1**), in which the azabicyclooctane moiety is replaced by diazabicyclooctane or diazabicyclononane, were synthesized and their infectivity reduction power was determined through a viral neutralization assay on a panel of six pseudoviruses. Diazabicyclooctane **2** demonstrated a biological activity similar to that of maraviroc (**1**), whereas diazabicyclononane **3** showed a lower infectivity reduction.

To explain these results, conformational analyses, NMR and docking experiments were performed. The modeling study revealed that maraviroc and its derivatives are highly flexible molecules with several degrees of conformational freedom, and high-field 1D and 2D NMR experiments confirmed this hypothesis showing the existence in solution of the calculated conformers as evidenced by specific NOESY contacts. Actually, from a conformational point of view, no significant differences were observed between the corresponding conformations of the three compounds; however, they could be divided into two families depending on the orientation of the isopropyl group with respect to the ethylene bridge (A, B, E, F, G, H *vs.* C, D, I, see *τ*_7_ values in Tables S1 and 2, ESI‡) and different percentages of these families were observed for the three compounds.

The above-mentioned crystal structure of the CCR5 maraviroc complex^5^ shows that the reference compound **1** orients the isopropyl group on the opposite side of the ethylene bridge of the bicyclic system, indicating this conformation probably as the most active. These data seem to be in contrast to our results that show compound **1** (see Fig. 2) having just about 14% of the active conformations C and D (Table S1‡) with respect to the less active **2** having about 42% of these favorable conformations (Table S2‡). However, we can observe that the low rotational barrier around *τ*_7_ (12.41 kcal mol^–1^, Table S1‡) of **1** might make the interconversion of all its conformers into the most active ones easy when interacting with CCR5. Conversely, analogue **2** has an almost twice as large barrier value (20.09 kcal mol^–1^, Table S2‡) that could hamper the interconversion reducing the amount of the active conformers during the interaction with the protein.

Using the same criterion, the lower *τ*_7_ rotational barrier of **3** (16.74 kcal mol^–1^, Table S2‡) with respect to **2** should provide a higher activity of **3***vs.***2**. Thus, its observed lower activity (see Fig. 2) could be due to the different bridge length.

Docking calculations were useful for shedding some light on the molecular interactions participated in by the tropane ring moiety of maraviroc when bound to CCR5. By evaluating the role of W1220 in favoring or not the theoretical affinity of compounds **2** and **3**, we suggested that the homologation of the tropane ring (as in the structure of compound **3**) was detrimental to a fruitful interaction of the ligand with the CCR5 binding site.

In conclusion, in this work, the importance of the rotational barrier around *τ*_7_ of maraviroc and the relevance of the bridge length of its bicyclic scaffold have been proposed as critical structural features able to modulate its anti-viral activity laying the foundations for the synthesis of improved active compounds.

## Experimental

### Chemistry

Reagents were purchased from commercial suppliers and used as-received. Commercial plates on aluminum backed silica gel 60 plates (0.2 mm, Merck) were used for analytical TLC to monitor the course of the reaction. Silica gel 60 (Merck 40–63 μm) was used for flash chromatography to purify the intermediates and final compounds. The purity of the final compounds was determined by HPLC analysis and was ≥95%. Melting points were determined in open capillary tubes with a Büchi Melting Point 510. ^1^H NMR spectra were acquired at ambient temperature with a 300 MHz Oxford-Varian instrument. Chemical shifts are expressed in ppm from tetramethylsilane resonance in the indicated solvent (TMS: *δ* = 0.0 ppm). Derivatives **4** and **5** were synthesized according to literature methods.^19^ The structures of all compounds are consistent with their analytical and spectroscopic data.

#### Synthesis of maraviroc (**1**)

Maraviroc (**1**) was prepared according to a literature procedure.^18^^1^H-NMR: see Table S3.‡ ^13^C-NMR (CD_3_OD): *δ* = 10.4 (*C*H_3_), 20.1 (2 *C*H_3_ iPr), 24.7 (C5′′′), 24.8 (C6 and C7), 25.0 (C6′′ or C2′′, d, *J*_C–F_ = 9.5 Hz), 25.2 (C2′′ or C6′′, d, *J*_C–F_ = 9.5 Hz), 31.8–31.9 (C3′′ and C5′′, t, *J*_C–F_ = 25 Hz), 33.7 (C8b), 34.6 (C2 and C4), 41.7 (C1′′), 47.2 (C3), 47.9 (C8a), 50.7 (C8c), 58.4 (C1 or C5), 59.2 (C5 or C1), 121.9 (C4′′, t, *J*_C–F_ = 240 Hz), 125.7 (C2′ and C6′), 126.4 (C4′), 127.7 (C3′ and C5′), 141.7 (C1′), 150.6 (C5′′′ or C2′′′), 159.4 (C2′′′ or C5′′′) and 174.9 (C1′′a). Experimental: p*K*_a1_ = 7.919; p*K*_a2_ = 3.443 (see the ESI‡).

#### General procedure A for the synthesis of methyl-3-benzyl-3,8-diazabicyclo[3.2.1]octane-8-carboxylate (**6**) and methyl-3-benzyl-3,9-diazabicyclo[3.3.1]nonane-9-carboxylate (**7**)

To a cooled solution of the appropriate diazabicyclo derivatives (**4**, **5**; 8.75 mmol) and triethylamine (1.83 mL, 13 mmol) in diethyl ether (50 mL), methyl chloroformate (1.01 mL, 13 mmol) was slowly added and the mixture was stirred overnight at room temperature. After completion of the reaction, the mixture was washed with 5% NaHCO_3_ solution (20 mL), dried over anhydrous Na_2_SO_4_, and filtered and the solvent was removed under reduced pressure to give the final products.

#### Methyl-3-benzyl-3,8-diazabicyclo[3.2.1]octane-8-carboxylate (**6**)

General procedure A was followed for the synthesis of compound **6**, which was obtained as a pale yellow oil after purification by flash column chromatography (cyclohexane/ethyl acetate, 80 : 20); 88% yield. TLC (cyclohexane/ethyl acetate, 80 : 20): Rf = 0.45. ^1^H-NMR (300 MHz, acetone-d_6_) *δ* (ppm): 1.70–1.95 (m, 4H, 2H_6_, 2H_7_); 2.15–2.30 (m, 2H, 2H_2_ (H_4_)); 2.55–2.65 (m, 2H, 2H_4_ (H_2_)); 3.45 (s, 2H, CH_2_Ph); 3.60 (s, 3H, OCH_3_); 4.10–4.20 (m, 2H, H_1_, H_5_); 7.15–7.40 (m, 5H, Ph).

#### Methyl-3-benzyl-3,9-diazabicyclo[3.3.1]nonane-9-carboxylate (**7**)

General procedure A was followed for the synthesis of compound **7**, which was obtained as an orange oil after purification by flash column chromatography (cyclohexane/ethyl acetate, 90 : 10); 69% yield. TLC (petroleum ether/ethyl acetate, 90 : 10): Rf = 0.3. ^1^H-NMR (300 MHz, CDCl_3_) *δ* (ppm): 1.50–1.90 (m, 5H, 2H_6_, H_7_, 2H_8_); 2.25–2.40 (m, 2H, H_7_, H_2_ (H_4_)); 2.75–2.95 (m, 3H, H_2_ (H_4_), 2H_4_ (H_2_)); 3.40 (s, 2H, CH_2_Ph); 3.70 (s, 3H, OCH_3_); 4.05–4.25 (m, 2H, H_1_, H_5_); 7.20–7.40 (m, 5H, Ph).

#### General procedure B for the synthesis of methyl-3,8-diazabicyclo[3.2.1]octane-8-carboxylate (**8**) and methyl-3,9-diazabicyclo[3.3.1]nonane-9-carboxylate (**9**)

The appropriate diazabicyclo derivatives (**6**, **7**) were dissolved in ethanol (10 mL), 10% Pd–C (10% in weight) was added to the solution, and the mixture was hydrogenated at room temperature under external pressure. After the uptake of hydrogen ceased, the catalyst was filtered off, and the solvent was evaporated to give final compounds.

#### Methyl-3,8-diazabicyclo[3.2.1]octane-8-carboxylate (**8**)

General procedure B was followed for the synthesis of compound **8**, which was obtained as a yellow oil without further purification (99% yield). TLC (dichloromethane/methanol, 85 : 15): Rf = 0.36. ^1^H-NMR (300 MHz, CDCl_3_) *δ* (ppm): 1.65–1.85 (m, 4H, 2H_6_, 2H_7_); 2.50–2.60 (m, 2H, 2H_2_ (H_4_)); 2.75–2.95 (m, 2H, 2H_4_ (H_2_)); 3.55 (s, 3H, OCH_3_); 3.95–4.10 (m, 2H, H_1_, H_5_).

#### Methyl-3,9-diazabicyclo[3.3.1]nonane-9-carboxylate (**9**)

General procedure B was followed for the synthesis of compound **9**, which was obtained as a yellow oil without further purification (91% yield). TLC (dichloromethane/methanol, 85 : 15): Rf = 0.35. ^1^H-NMR (300 MHz, CDCl_3_) *δ* (ppm): 1.50–2.80 (m, 6H, 2H_6_, H_7_, 2H_8_, NH); 2.40–2.55 (m, 1H, H_7_); 2.90–3.10 (m, 4H, H_2_, H_4_); 3.65 (s, 3H, OCH_3_); 3.90–4.15 (m, 2H, H_1_, H_5_).

#### General procedure C for the synthesis of methyl-3-nitroso-3,8-diazabicyclo[3.2.1]octane-8-carboxylate (**10**) and methyl-3-nitroso-3,9-diazabicyclo[3.3.1]nonane-9-carboxylate (**11**)

To a stirred and cooled solution of **8** or **9** (4.86 mmol) in 2 M hydrochloric acid (25 mL), a solution of sodium nitrite (20 mmol) in 5 mL of water was added dropwise under nitrogen. The mixture was stirred for 4 h at room temperature, then cooled, made alkaline with 50% sodium hydroxide, and extracted with ethyl ether. The extract was dried over Na_2_SO_4_, the solvent evaporated, and the residue was purified by flash chromatography to give the desired products **10** or **11**.

#### Methyl-3-nitroso-3,8-diazabicyclo[3.2.1]octane-8-carboxylate (**10**)

General procedure C was followed for the synthesis of compound **10**, which was obtained as a yellow oil after purification by flash column chromatography (petroleum ether/ethyl acetate, 60 : 40); 75% yield. TLC (petroleum ether/ethyl acetate, 60 : 40): Rf = 0.2. ^1^H-NMR (300 MHz, CDCl_3_) *δ* (ppm): 1.36–2.21 (m, 4H, 2H_6_, 2H_7_); 2.76–2.83 (m, 1H, H_2_ (H_4_)); 3.75 (s, 3H, OCH_3_); 3.97–4.04 (m, 1H, H_2_ (H_4_)); 4.34–4.40 (m, 1H, H_5_); 4.51–4.60 (m, 2H, H_1_, H_4_ (H_2_)); 4.76–4.84 (m, 1H, H_4_ (H_2_)).

#### Methyl-3-nitroso-3,9-diazabicyclo[3.3.1]nonane-9-carboxylate (**11**)

General procedure C was followed for the synthesis of compound **11**, which was obtained as a yellow oil after purification by flash column chromatography (petroleum ether/ethyl acetate, 50 : 50); 51% yield. TLC (petroleum ether/ethyl acetate, 60 : 40): Rf = 0.33. ^1^H-NMR (300 MHz, CDCl_3_) *δ* (ppm): 1.44–2.00 (m, 6H, 2H_6_, 2H_7_, 2H_8_); 2.74–2.89 (m, 1H, H_2_ (H_4_)); 3.78 (s, 3H, OCH_3_); 3.96–4.09 (m, 1H, H_2_ (H_4_)); 4.32–4.66 (m, 2H, H_1_, H_5_); 4.74–5.00 (m, 2H, 2H_4_ (H_2_)).

#### General procedure D for the synthesis of methyl-3-amino-3,8-diazabicyclo[3.2.1]octane-8-carboxylate (**12**) and methyl-3-amino-3,9-diazabicyclo[3.3.1]nonane-9-carboxylate (**13**)

To a stirred suspension of zinc dust (1.81 mmol) in 1.9 mL of 1 : 1 acetic acid–water, a solution of **10** or **11** (0.4 mmol) in 1.3 mL of acetic acid was added under nitrogen, maintaining the temperature between 10 and 20 °C. The mixture was stirred for 15 min at room temperature and then heated to 80 °C for 15 min. After 5 min of heating, an additional portion of zinc dust (1.21 mmol) was added. The hot solution was then filtered and the zinc and inorganic salts were washed with three 1.5 mL portions of hot 1 N hydrochloric acid. The combined filtrate and washings were basified with 50% aqueous sodium hydroxide solution and extracted with dichloromethane (3 × 10 mL). The combined organic extracts were dried over anhydrous Na_2_SO_4_ and evaporated. Compounds **12** and **13** were not purified or further characterized due to their instability, and immediately used for the next reactions. Compound **12** was a yellow oil: TLC (dichloromethane/methanol, 95 : 5): Rf = 0.2. Compound **13** was a yellow oil: TLC (dichloromethane/methanol, 93 : 7): Rf = 0.3.

#### General procedure E for the synthesis of methyl-3-isobutyramido-3,8-diazabicyclo[3.2.1]octane-8-carboxylate (**14**) and methyl-3-isobutyramido-3,9-diazabicyclo[3.3.1]nonane-9-carboxylate (**15**)

To a solution of the appropriate amino derivatives (**12**, **13**; 2.43 mmol) in dimethylformamide (1 mL), triethylamine (4.01 mmol), isobutyric acid (3.95 mmol) and HATU (2.39 mmol) were added. The resultant mixture was stirred for 17 h at room temperature. Finally, water (3 mL) and ethyl acetate (3 mL) were added and the phases separated. The organic layer was extracted with 6 M HCl (3 × 5 mL) and then a NaOH pellet was added to the cooled aqueous acidic phase until pH 9–10. The basic layers were extracted with ethyl acetate (3 × 10 mL). The combined organic extracts were dried over anhydrous Na_2_SO_4_ and evaporated under reduced pressure to give the final products.

#### Methyl-3-isobutyramido-3,8-diazabicyclo[3.2.1]octane-8-carboxylate (**14**)

General procedure E was followed for the synthesis of compound **14**, which was obtained as a yellow oil after purification by flash column chromatography (dichloromethane/methanol, 95 : 5); 60% yield from **10**. TLC (dichloromethane/methanol, 95 : 5): Rf = 0.28. ^1^H-NMR (300 MHz, CDCl_3_) *δ* (ppm): 1.11–1.18 (m, 6H, 2CH_3_); 1.96–2.17 (m, 4H, 2H_6_, 2H_7_); 2.60–2.68 (m, 1H, H_2_ (H_4_)); 2.87–2.95 (m, 3H, H_2_ (H_4_), 2H_4_ (H_2_)); 3.00–3.15 (m, 1H, C*H*(CH_3_)_2_); 3.71 (s, 3H, OCH_3_); 4.20–4.38 (m, 2H, H_1_, H_5_).

#### Methyl-3-isobutyramido-3,9-diazabicyclo[3.3.1]nonane-9-carboxylate (**15**)

General procedure E was followed for the synthesis of compound **15** which was obtained as a yellow oil after purification by flash column chromatography (dichloromethane/methanol, 95 : 5); 60% yield from **11**. TLC (dichloromethane/methanol, 85 : 15): Rf = 0.23. ^1^H-NMR (300 MHz, CDCl_3_) *δ* (ppm): 1.07–1.17 (m, 6H, 2CH_3_); 1.48–1.94 (m, 5H, 2H_6_, H_7_, 2H_8_); 2.40–2.60 (m, 1H, H_7_); 2.60–2.75 (m, 2H, 2H_2_ (H_4_)); 2.86–3.11 (m, 3H, C*H*(CH_3_)_2_, 2H_4_ (H_2_)); 3.68 (s, 3H, OCH_3_); 4.11–4.31 (m, 2H, H_1_, H_5_).

#### General procedure F for the synthesis of methyl-3-(3-isopropyl-5-methyl-4*H*-1,2,4-triazol-4-yl)-3,8-diazabicyclo[3.2.1]octane-8-carboxylate (**16**) and methyl-3-(3-isopropyl-5-methyl-4*H*-1,2,4-triazol-4-yl)-3,9-diazabicyclo[3.3.1]nonane-9-carboxylate (**17**)

A mixture of dichloromethane (4.8 mL) and PCl_5_ (2.7 mmol) was cooled to –5 °C. A solution of butyramido derivatives **16** or **17** (2.08 mmol) in dichloromethane (1.6 mL) was slowly added keeping the temperature below 10 °C. The solution was warmed to ambient temperature and held at this temperature for 2.5 h, then cooled back to –5 °C. A solution of acetic hydrazide (3.33 mmol) in 2-methyl-2-butanol was prepared by dissolving acetic hydrazide in acetonitrile (1.8 mL) and 2-methyl-2-butanol (9.8 mL), and then concentrating it to approximately half volume by distillation under reduced pressure. The acetic hydrazide solution was added to the reaction mixture keeping the temperature below 10 °C. The resultant solution was warmed to ambient temperature and stirred for 19 h. The mixture was cooled to –5 °C and 2 M NaOH (3 mL) was added, keeping the temperature below 10 °C, and the layers were separated. The organic layer was concentrated under reduced pressure. The residue was treated with 2-methyl-2-butanol (1.5 mL) and acetic acid (0.2 mL) and warmed at 80 °C for 2.5 h. The solution was cooled to 0 °C and 2 M NaOH was added until pH 12. The phases were separated and the aqueous layer was extracted with ethyl acetate (3 × 15 mL). The combined organic extracts were dried over anhydrous Na_2_SO_4_ and evaporated under reduced pressure to give the final products.

#### Methyl-3-(3-isopropyl-5-methyl-4*H*-1,2,4-triazol-4-yl)-3,8-diazabicyclo[3.2.1]octane-8-carboxylate (**16**)

General procedure F was followed for the synthesis of compound **16**, which was obtained as an orange oil after purification by flash column chromatography (petroleum ether/ethyl acetate, 4 : 6); 48% yield. TLC (dichloromethane/methanol, 9 : 1): Rf = 0.38. ^1^H-NMR (300 MHz, CDCl_3_) *δ* (ppm): 1.34–1.40 (m, 6H, 2CH_3_); 2.00–2.05 (m, 4H, 2H_6_, 2H_7_); 2.35–2.51 (m, 3H, CH_3_); 2.88–2.95 (m, 2H, 2H_2_ (H_4_)); 3.03–3.18 (m, 1H, C*H*(CH_3_)_2_); 3.44–3.56 (m, 2H, 2H_4_ (H_2_)); 3.76 (s, 3H, OCH_3_); 4.38–4.48 (m, 2H, H_1_, H_5_).

#### Methyl-3-(3-isopropyl-5-methyl-4*H*-1,2,4-triazol-4-yl)-3,9-diazabicyclo[3.3.1]nonane-9-carboxylate (**17**)

General procedure F was followed for the synthesis of compound **17**, which was obtained as a white solid after purification by flash column chromatography (dichloromethane/methanol, 95 : 5); 48% yield. M.p. 176–176.8 °C. TLC (dichloromethane/methanol, 9 : 1): Rf = 0.26. ^1^H-NMR (300 MHz, CDCl_3_) *δ* (ppm): 1.36–1.40 (m, 6H, 2CH_3_); 1.66–2.05 (m, 5H, 2H_6_, H_7_, 2H_8_); 2.40–2.59 (m, 3H, CH_3_); 2.59–2.73 (m, 1H, H_7_); 3.05–3.19 (m, 3H, C*H*(CH_3_)_2_, 2H_2_ (H_4_)); 3.61–3.73 (m, 2H, 2H_4_ (H_2_)); 3.80 (s, 3H, OCH_3_); 4.31–4.53 (m, 2H, H_1_, H_5_).

#### General procedure G for the synthesis of 3-(3-isopropyl-5-methyl-4*H*-1,2,4-triazol-4-yl)-3,8-diazabicyclo[3.2.1]octane (**18**) and 3-(3-isopropyl-5-methyl-4*H*-1,2,4-triazol-4-yl)-3,9-diazabicyclo[3.3.1]nonane (**19**)

To a solution of the appropriate triazole derivatives (**16** or **17**, 0.17 mmol) in methanol (2.9 mL), 10% NaOH was added (2.9 mL). The solution was warmed at 80 °C for 5 h. The methanol was removed under reduced pressure and dichloromethane (10 mL) was added. The phases were separated, and the organic phase was dried over anhydrous Na_2_SO_4_ and evaporated under reduced pressure to give the final products.

#### 3-(3-Isopropyl-5-methyl-4*H*-1,2,4-triazol-4-yl)-3,8-diazabicyclo[3.2.1]octane (**18**)

General procedure G was followed for the synthesis of compound **18**, which was obtained as a pale yellow oil and was utilized without further purification (57% yield). TLC (dichloromethane/methanol, 7 : 3): Rf = 0.18. ^1^H-NMR (300 MHz, CDCl_3_) *δ* (ppm): 1.32–1.36 (m, 6H, 2CH_3_); 1.80–1.86 (m, 2H, 2H_6_ (H_7_)); 1.96–2.14 (m, 2H, 2H_7_ (H_6_)); 2.32–2.54 (m, 3H, CH_3_); 2.84–2.90 (m, 2H, 2H_2_ (H_4_)); 3.01–3.18 (m, 1H, C*H*(CH_3_)_2_); 3.44–3.50 (m, 2H, 2H_4_ (H_2_)); 3.58–3.64 (m, 2H, H_1_, H_5_).

#### 3-(3-Isopropyl-5-methyl-4*H*-1,2,4-triazol-4-yl)-3,9-diazabicyclo[3.3.1]nonane (**19**)

General procedure G was followed for the synthesis of compound **19**, which was obtained as a yellow foam and was utilized without further purification (98% yield). TLC (dichloromethane/methanol, 7 : 3): Rf = 0.11. ^1^H-NMR (300 MHz, CDCl_3_) *δ* (ppm): 1.28–1.37 (m, 6H, 2CH_3_); 1.63–2.00 (m, 5H, 2H_6_, H_7_, 2H_8_); 2.34–2.51 (m, 3H, CH_3_); 2.51–2.68 (m, 1H, H_7_); 2.97–3.14 (m, 2H, C*H*(CH_3_)_2_, H_2_ (H_4_)); 3.18–3.28 (m, 1H, H_2_ (H_4_)); 3.55–3.71 (m, 2H, 2H_4_ (H_2_)); 4.20–4.44 (m, 2H, H_1_, H_5_).

#### Synthesis of (*S*)-4,4-difluoro-*N*-(3-oxo-1-phenylpropyl)cyclohexanecarboxamide (**20**)

Compound **20** was synthesized following the procedure reported in the literature^20^ and its analytical data were comparable to those reported. White solid. M.p. 119 °C. TLC (petroleum ether/ethyl acetate, 4 : 6): Rf = 0.52. ^1^H-NMR (300 MHz, CDCl_3_) *δ* (ppm): 1.60–2.24 (m, 8H); 2.92–3.12 (m, 2H); 5.44–4.55 (m, 1H); 6.20–6.26 (d, br, 1H); 7.20–7.40 (m, 5H); 9.75 (s, 1H). *m*/*z* 296.07 [M + H]^+^.

#### Synthesis of 4,4-difluoro-*N*-((1*S*)-3-(3-(3-isopropyl-5-methyl-4*H*-1,2,4-triazol-4-yl)-3,8-diazabicyclo[3.2.1]octan-8-yl)-1-phenylpropyl)cyclohexanecarboxamide (**2**)

To a suspension of **18** (22.8 mg, 0.097 mmol) in dichloromethane (0.4 mL) under a nitrogen atmosphere, a solution of **20** (31 mg, 0.106 mmol) in toluene (0.5 mL) and acetic acid (0.01 mL) were added. The solution was stirred for 30 min at room temperature, then it was cooled at 0 °C and sodium triacetoxyborohydride (25 mg, 0.116 mmol) was added. The resultant mixture warmed to room temperature was stirred for further 4 h. Finally, the reaction was quenched with water (3 mL) and the pH was adjusted to 11–12 by addition of 2 M sodium hydroxide solution. The phases were separated and the organic phase was dried over anhydrous Na_2_SO_4_ and evaporated under reduced pressure to give a yellow oil that was purified by flash column chromatography (dichloromethane/methanol, 9 : 1); 37.7% yield. TLC (dichloromethane/methanol, 9 : 1): Rf = 0.24. ^1^H-NMR: see Table S3.‡ ^13^C-NMR (CD_3_OD): *δ* = 8.0 (*C*H_3_), 10.6 (*C*H_3_), 19.3 (2 *C*H_3_ iPr), 20.2 (2 *C*H_3_ iPr), 23.1 (C6 and C7), 24.0 (C5′′′a), 24.7 (C5′′′a), 25.0–25.1 (C2′′ and C6′′, d, *J*_C–F_ = 9.5 Hz), 30.0 (C8b), 31.8 (C3′′ and C5′′, t, *J*_C–F_ = 25 Hz), 41.4 (C1′′), 48.6–48.7 (C8a), 50.1 (C8c), 54.7–54.9 (C2 and C4), 61.3–61.5 (C1 or C5), 61.9 (C5 or C1), 121.9 (C4′′, t, *J*_C–F_ = 240 Hz), 125.8 (C2′ and C6′), 127.0 (C4′), 128.0 (C3′ and C5′), 140.1 (C1′), 151.2 (C5′′′ or C2′′′), 159.9 (C2′′′ or C5′′′) and 175.7 (C1′′a); [*α*]25D = –20.05 (*c* = 6.3 × 10^–3^ M, CHCl_3_). ESI-HRMS *m*/*z*: [M + H]^+^ measured: 515.33088; calculated: 515.33044; [M + Na]^+^ measured: 537.31234; calculated: 537.31239. Experimental: p*K*_a1_ = 7.268; p*K*_a2_ = 2.958 (see the ESI‡).

#### Synthesis of 4,4-difluoro-*N*-((1*S*)-3-(3-(3-isopropyl-5-methyl-4*H*-1,2,4-triazol-4-yl)-3,9-diazabicyclo[3.3.1]nonan-9-yl)-1-phenylpropyl)cyclohexanecarboxamide (**3**)

To a suspension of **19** (47 mg, 0.187 mmol) in dichloromethane (0.7 mL) under a nitrogen atmosphere, a solution of **20** (60 mg, 0.204 mmol) in toluene (1 mL) and acetic acid (0.02 mL) were added. The solution was stirred for 3 h at room temperature, then it was cooled at 0 °C and sodium triacetoxyborohydride (48 mg, 0.224 mmol) was added. The resultant mixture was warmed to room temperature and stirred for further 4 h. Finally, the reaction was quenched with water (4 mL) and the pH was adjusted to 11–12 by addition of 2 M sodium hydroxide solution. The phases were separated and the organic layer was dried over anhydrous Na_2_SO_4_ and evaporated under reduced pressure to give a white foam that was purified by flash column chromatography (dichloromethane/methanol, 97 : 3); 21.3% yield. TLC (dichloromethane/methanol, 9 : 1): Rf = 0.45. ^1^H-NMR: see Table S3.‡ ^13^C-NMR (CD_3_OD : D_2_O, 70 : 30): *δ* = 9.1 (*C*H_3_), 11.4 (*C*H_3_), 17.0 (C7), 19.1 (2 *C*H_3_ iPr), 20.3 (2 *C*H_3_ iPr), 23.2 (C6 and C7), 24.2 (C5′′′a), 24.8–24.9 (C2′′ and C6′′, d, *J*_C–F_ = 9.5 Hz), 30.0 (C9b), 31.5 (C3′′ and C5′′, t, *J*_C–F_ = 25 Hz), 41.3 (C1′′), 47.50 (C9a), 50.5 (C9c), 52.5 (C2 and C4), 52.8 (C1 or C5), 53.4 (C5 or C1), 122.3 (C4′′, t, *J*_C–F_ = 240 Hz), 125.7 (C2′ and C6′), 127.0 (C4′), 128.0 (C3′ and C5′), 140.0 (C1′), 150.1 (C5′′′ or C2′′′), 159.7 (C2′′′ or C5′′′) and 175.6 (C1′′a); [*α*]25D = –23.34 (*c* = 6.3 × 10^–3^ M, CHCl_3_). ESI-HRMS *m*/*z*: [M + H]^+^ measured: 529.34671; calculated: 529.34609; [M + Na]^+^ measured: 551.32845; calculated: 551.32804. Experimental: p*K*_a1_ = 7.155; p*K*_a2_ = 2.923 (see the ESI‡).

### Generation and titration of virus stocks

293 T cell-derived stocks of pseudoviruses were generated by proviral DNA transfection using FuGENE 6, according to the manufacturer's protocol (Promega, Madison, WI). Viral supernatants were harvested 72 h post transfection, clarified at 1800 rpm for 20 min, and frozen at –70 °C. The virus stocks were further analyzed for firefly luciferase expression in the TZM-bl cell line. Four replicates of five-fold dilutions of viruses were added to 96 flat-bottomed plate wells containing 10^4^ TZM-bl cells per well, in 10% D-MEM growth medium with 7.5 μg ml^–1^ DEAE-dextran (Sigma) in a final volume of 200 μl. After 48 h of incubation at 37 °C, 100 μL of culture medium was removed from each well and replaced with 100 μL of Bright-Glo luciferase reagent (Promega). After 2 min of incubation, 150 μL of the cell lysate was transferred to a 96-well white solid plate and luminescence was measured using a Victor Light 2030 luminometer (Perkin Elmer). Fifty percent infectious dose (ID50) titers were defined as the reciprocal of the virus dilution yielding 50% positive wells (Reed–Muench calculation).

### Viral neutralization assay

Infectivity reduction was measured as a reduction in Luc reporter gene expression after a single round of virus infection in TZM-bl cells with env-pseudotyped viruses.^17^–^30^ The virus panel of HIV-related pseudoviruses included one laboratory strain SF162 (Clade B, Tier 1 virus), four primary infected Subtype B subjects such as QH0692, 6535, PVO, and AC10 (all strains were CCR5-tropic, Tier 2), and one primary infected Subtype C virus, ZM214 (CCR5-tropic, Tier 2). In order to demonstrate the specificity of HIV neutralization, two HIV-unrelated viruses (VSV-G virus, strain SVA.MLV#922, an unrelated strain) and one CXCR4 tropic peseudovirus (HXB2, Tier 1) were also included. Briefly, 200 TCID50 of pseudoviruses in 50 μL of culture media were incubated with 100 μL of serially diluted compounds (from 10 to 0.001 μM) by using D-MEM with 10% foetal bovine serum in a 96-well plate for 1 h at 37 °C. Compound **1**, maraviroc, was used as the positive control. A 100 μL solution of TZM-bl cells (10^4^ cells per well) containing 15 μg mL^–1^ DEAE dextran was added; the cultures were then incubated at 37 °C in 5% CO_2_/95% air for 48 h and 72 h. Assay controls included replicate wells of TZM-bl cells alone (cell control) and TZM-bl cells with virus (virus control). Four replicates have been performed per each point. Infection was monitored by evaluating the luciferase activity. Infectivity reduction was calculated as IC_90_, the compound concentration at which relative luminescence units (RLU) were reduced by 90% relative to virus control wells (wells with no inhibitor) after subtraction of background RLU in the cell control wells (wells without virus infection).

### Proliferation assays

All assays were performed in 96-well microtiter plates. To each well were added 10^4^ TZM-bl cells and the highest concentration of the compounds used for neutralization assay (10 μM) was tested for all three compounds and added after cells were seeded to wells, corresponding to time 0. The cells were allowed to proliferate for 48 and 72 h at 37 °C in a humidified CO_2_-controlled atmosphere. At the end of the incubation period, the cells were counted in a Coulter counter (Scepter™ 2.0). 100% of cell proliferation corresponds to 100 000 and 150 000 cells after 48 h and 72 h of cell incubation with medium and without compounds, respectively. Cells were also treated with Trypan Blue to be sure that all compounds did not induce cellular toxicity.

### Conformational analysis

A systematic search of the conformational space of compound **1** was performed by using the Gaussian09 program package^31^ through optimizations in the gas phase at the B3LYP/6-31G(d) level.^23^ First, a starting geometry was constructed and optimized. Then, the energy profiles for rotation around the single bonds defined by *τ*_1_–*τ*_8_ were determined with a step size of 30° and the preferences of the difluorocyclohexane and piperidine rings were considered. All the combinations of the observed minima were used to generate all the possible starting geometries optimized as above. The procedure was similarly repeated for compounds **2** and **3**.

The conformational analysis of compounds **1–3** was also performed using Macromodel (v. 11.1) software.^32^ A total of 1000 conformations were generated and minimized for each compound using the OPLS_2005 force field^33^ with chloroform solvation and mixed torsional/low-mode sampling. Multiple searches were carried out using different starting geometries as well as different ring-closure bond choices. After completion of the search, the output files from the conformational searches were then analyzed using the XCluster program in MacroModel. Finally, structures with energy in the range of 4.0 kcal mol^–1^, higher than that of the current global minimum, were optimized at the DFT level (B3LYP/6-31G(d)).

Vibrational frequencies were computed at the same level of theory to verify that the optimized structures were minima. The solvent effects were considered by single-point calculations, at the same level as above, on the gas-phase optimized geometries, using a self-consistent reaction field (SCRF) method, based on the polarizable continuum model (PCM).^24^ The population percentages were calculated through the Boltzmann equation at 298 K.

### Docking calculations

The computational model of the CCR5 utilized for these studies was retrieved from the Protein Data Bank (PDB entry 4MBS).^34^ A chain of CCR5 was used in this study. Prior to starting the calculations, maraviroc, 1-oleoyl-*R*-glycerol and zinc ions found in the X-ray structure were removed. Moreover, the side chains of some residues and the hydrogen atoms not solved were added using the tleap module of Amber12.^35^ Ligands, in their ionized form, were docked in the protein area within 15 Å from the side chain of Trp248 and the calculations were performed using GOLD 5.2.2.^27^,^28^ Two hundred docking poses were generated and the ChemPLP scoring function was applied to rank the obtained solutions. All water molecules found in the binding site were considered in the docking runs. These water molecules were left free to rotate around their original position and, enabling the “toggle” option, we ensured that the optimal network of water molecules was considered to obtain the best docking pose for the ligands. Moreover, in order to evaluate the effect of the presence of Wat1220 on the docking score of the ligands, calculations were performed fixing or not the position of the solvent molecules and inactivating the “toggle” option for these molecules.

At the end of the docking calculations, all the generated poses were clustered by the complete-linkage method.^36^ This is a hierarchical-agglomerative clustering algorithm in which, initially, each element is located in a different singleton cluster. Then, clusters are combined into larger ones until all elements are in the same cluster. Finally, clusters displaying the imposed cutoff (RMSD values ≤ 2 Å) are selected and ranked depending on the ChemPLP score^37^ attained by the representative solution, the one with the highest score in each cluster.

Figures were generated by PyMOL Molecular Graphics System, version 1.6, Schrodinger, LLC (http://www.pymol.org).

### NMR spectroscopy

The NMR spectra of compounds **1–3** were recorded at 298 or 320 K with a Bruker AVANCE-500 spectrometer operating at 500.13 MHz for ^1^H or at 125.76 MHz for ^13^C NMR spectra using a 5 mm z-PFG (pulsed field gradient) broadband reverse probe. Chemical shifts are reported on the *δ* (ppm) scale and are relative to residual CH_3_OD at 3.30 ppm (central line) for ^1^H NMR spectra, and relative to CD_3_OD at 47.0 ppm (central line) for ^13^C NMR spectra. The data were collected and processed by XWIN-NMR software (Bruker) running on a PC with Microsoft Windows XP. Compounds (about 3–5 mg) were dissolved in the appropriate solvent (0.6 mL) which was CD_3_OD for **1** and **2** and CD_3_OD/D_2_O (70/30) for compound **3**, which gave very broad shaped signals in methanol only (see the ESI‡). The solutions were placed in a 5 mm NMR tube and the spectra were recorded at 298 K (compounds **1** and **2**) or 320 K (compound **3**). The signal assignment was given by a combination of 1D and 2D (COSY and HSQC) experiments, using standard Bruker pulse programs. The ^1^H–^1^H and ^13^C–^1^H bond correlations were confirmed by COSY and HSQC experiments using Z-PFGs. The pulse widths were 7.50 μs (90°) for ^1^H and 14.75 μs (90°) for ^13^C. Typically, 32 K data points were collected for one-dimensional spectra. The spectral width was 11.45 ppm (5733 Hz) for ^1^H NMR (digital resolution: 0.17 Hz per point). 2D experimental parameters were as follows. For ^1^H–^1^H correlations: relaxation delay 2.0 s, data matrix 1 K × 1 K (512 experiments to 1 K zero filling in F_1_, 1 K in F_2_), 2 or 24 transients in each experiment for COSY and NOESY, respectively, spectral width 8.00 ppm (3996 Hz). The NOESY spectra were generated with a mixing time of 1.0 s and acquired in the TPPI mode. There were no significant differences in the results obtained at different mixing times (0.5–1.5 s). For ^13^C–^1^H correlations (HSQC): relaxation delay 2.5 s, data matrix 1 K × 1 K (512 experiments to 1 K zero filling in F_1_, 1 K in F_2_), 6 transients in each experiment, spectral width 8.0 ppm (3996 Hz) in the proton domain and 150.0 ppm (18 864 Hz) in the carbon domain. A sinebell weighting was applied to each dimension. All 2D spectra were processed with the Bruker software package.

## Supplementary Material

Supplementary information
