# Supplementary material for: Diazabicyclo analogues of maraviroc: synthesis, modeling, NMR studies and antiviral activity
Source: Medchemcomm. 2016 Dec 16;8(2):422–33. doi: 10.1039/c6md00575f (PMC6071814; doi:10.1039/c6md00575f)
Supplement: Supplementary file 1 [file MD-008-C6MD00575F-s001.pdf]

Electronic Supplementary Material (ESI) for MedChemComm.
This journal is © The Royal Society of Chemistry 2016

Diazabicyclo Analogues of Maraviroc: Synthesis, Modeling, NMR Studies and Antiviral Activity

Laura Legnani, Diego Colombo, Lucia Lopalco, Assunta Venuti, Claudia Pastori, Lucio Toma, Matteo Mori, Giovanni Grazioso and Stefania Villa

SUPPLEMENTARY MATERIAL (A)

Page 2-10 .... ^{13}C NMR spectra of compounds **1**-**3**

Page 11- 18 .... ^{1}H NMR spectra of compounds **1**-**3**

Page 19.. Assignments of the axial and equatorial protons of 1-3

Page 20 .... Table S1. Relative energies (kcal/mol), equilibrium percentages at 298 K, and significant torsional angles[a] (°) of significantly populated conformers (P_i > 1%) of compound **1**.

Page 21 .... Figure S1. 3D-plots of conformers **C-H** of compound **1**.

Page 22 .... Table S2. Relative energies (kcal/mol), equilibrium percentages at 298 K, and torsional angles[a] (°) of significantly populated conformers (P_i > 1%) of compounds **2** and **3**.

Page 23-28 .... NOESY spectra of compounds **1**-**3**

Page 29 .... ^{1}H NMR spectra of compound **2** at increasing temperatures

Page 30 .... Table S3. ^{1}H NMR chemical shift (δ) of compounds **1**-**3**.

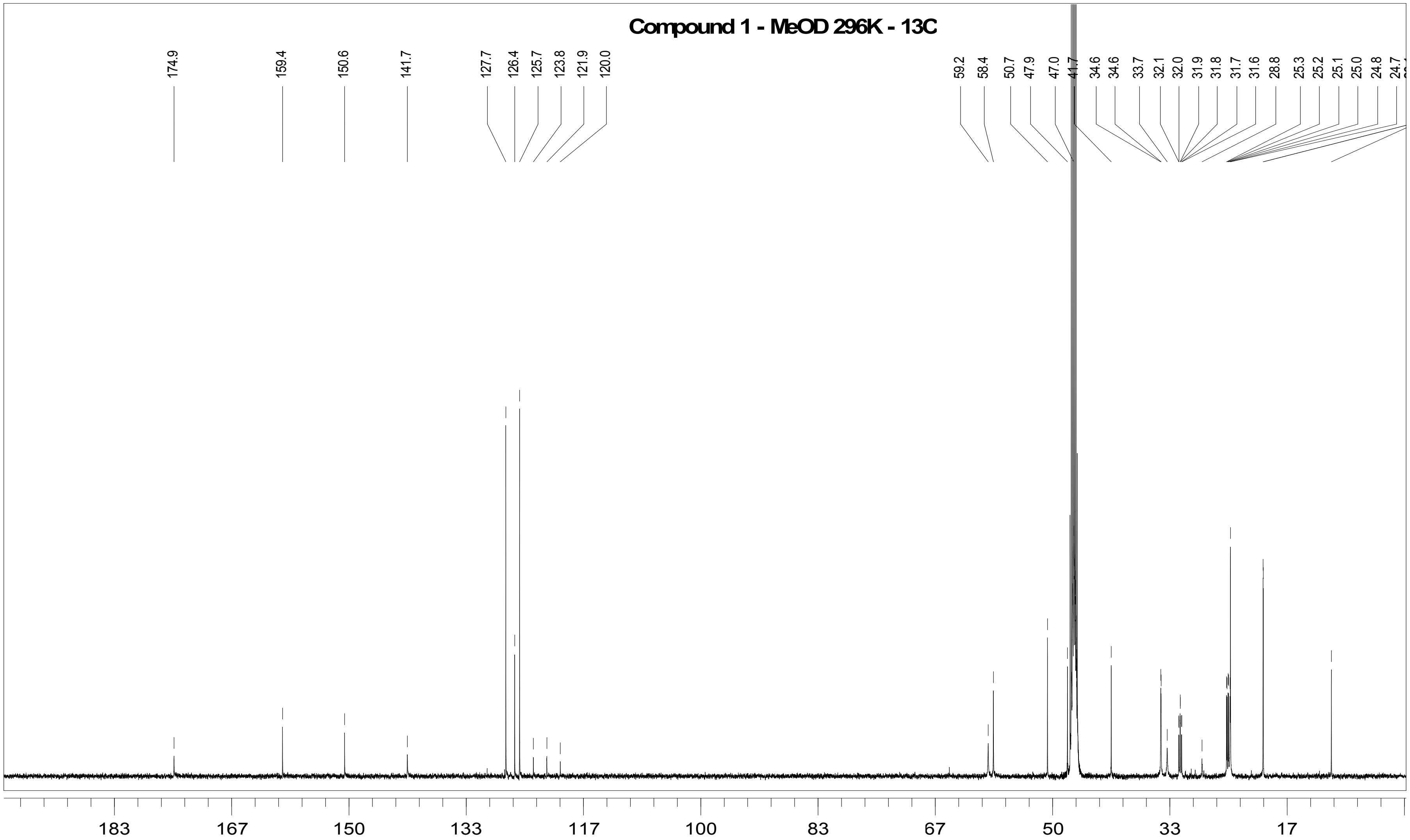
Compound 1 - MeOD 296K - 13C
174.9
159.4
150.6
141.7
127.7
126.4
125.7
123.8
121.9
120.0
59.2
58.4
50.7
47.9
47.0
41.7
34.6
34.6
33.7
32.1
32.0
31.9
31.8
31.7
31.6
28.8
25.3
25.2
25.1
25.0
24.8
24.7
183
167
150
133
117
100
83
67
50
33
17

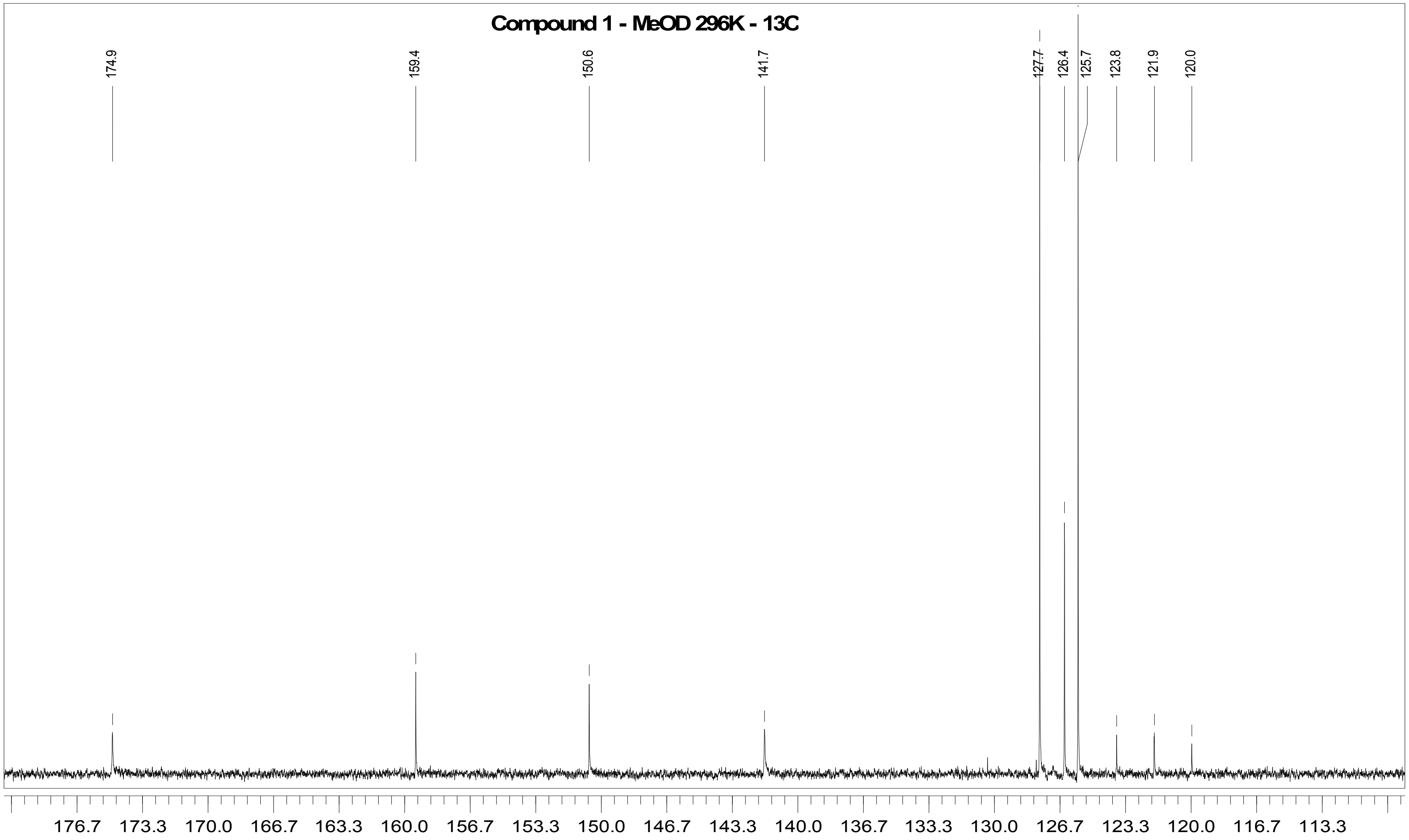
Compound 1 - MeOD 296K - 13C
174.9
159.4
150.6
141.7
127.7
126.4
125.7
123.8
121.9
120.0
176.7 173.3 170.0 166.7 163.3 160.0 156.7 153.3 150.0 146.7 143.3 140.0 136.7 133.3 130.0 126.7 123.3 120.0 116.7 113.3

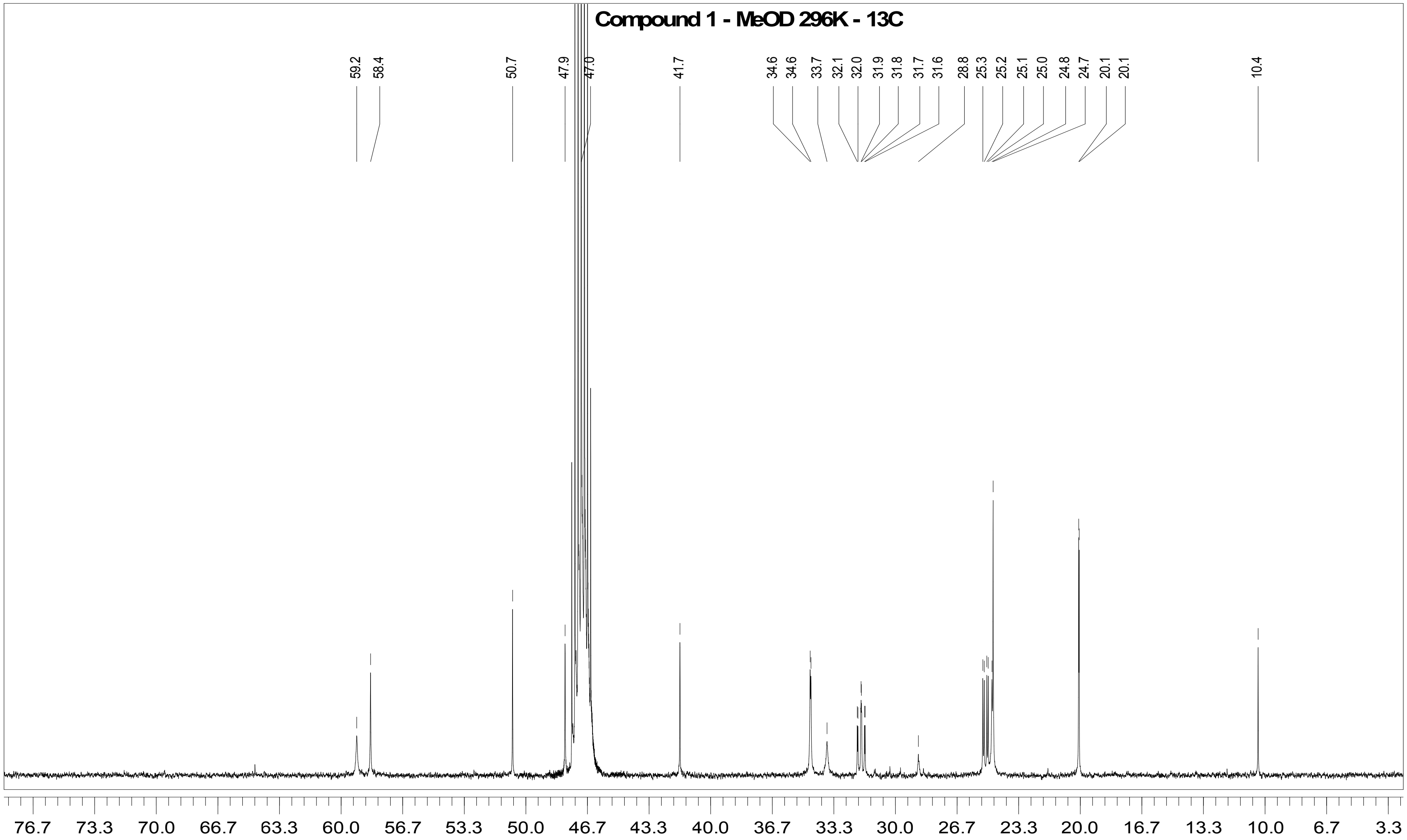
Compound 1 - MeOD 296K - 13C
59.2
58.4
50.7
47.9
47.0
41.7
34.6
34.6
33.7
32.1
32.0
31.9
31.8
31.7
31.6
28.8
25.3
25.2
25.1
25.0
24.8
24.7
20.1
20.1
10.4
76.7 73.3 70.0 66.7 63.3 60.0 56.7 53.3 50.0 46.7 43.3 40.0 36.7 33.3 30.0 26.7 23.3 20.0 16.7 13.3 10.0 6.7 3.3

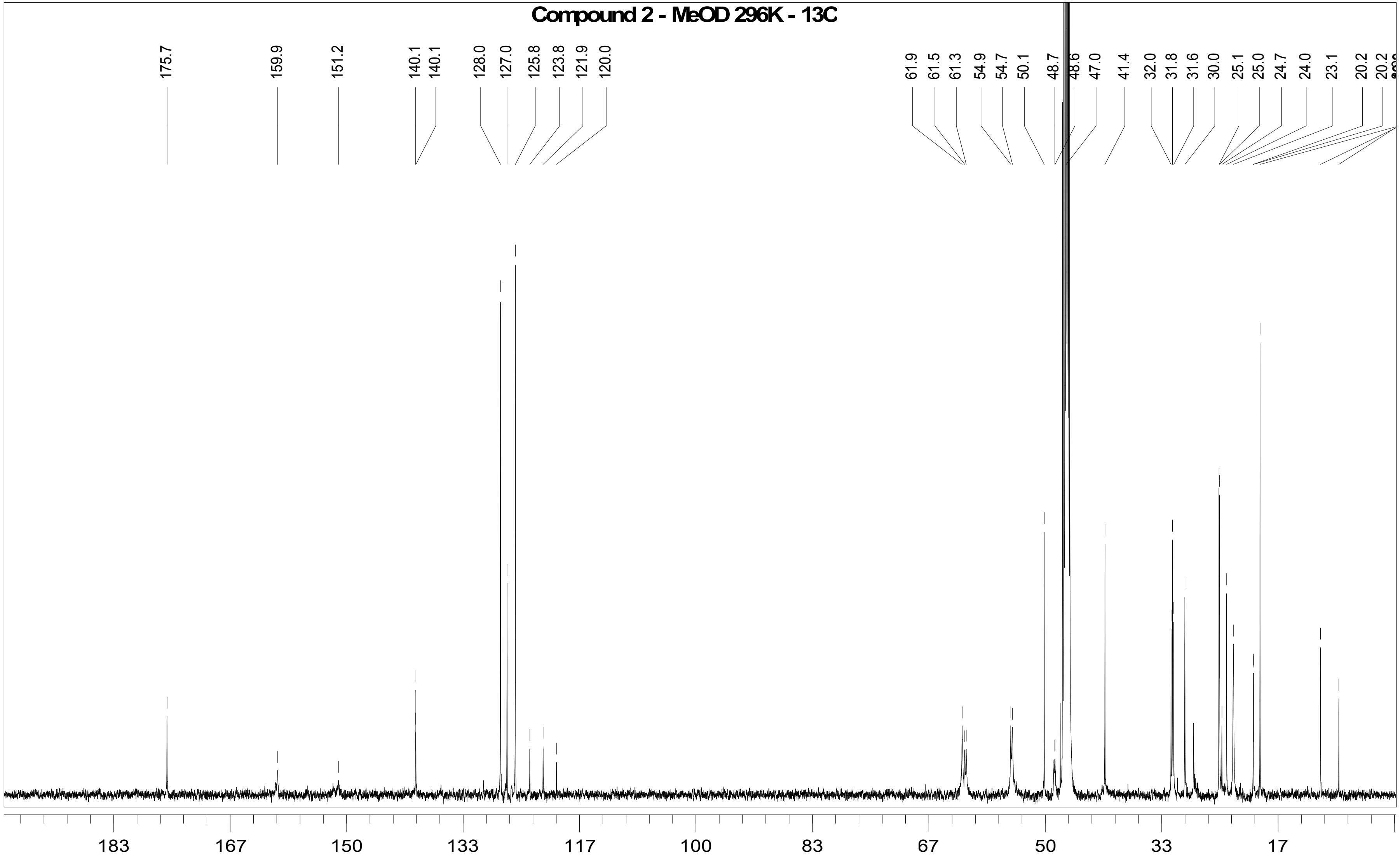

Compound 2 - MeOD 296K - 13C
175.7
159.9
151.2
140.1
140.1
128.0
127.0
125.8
123.8
121.9
120.0
61.9
61.5
61.3
54.9
54.7
50.1
48.7
48.6
47.0
41.4
32.0
31.8
31.6
30.0
25.1
25.0
24.7
24.0
23.1
20.2
20.2
183
167
150
133
117
100
83
67
50
33
17

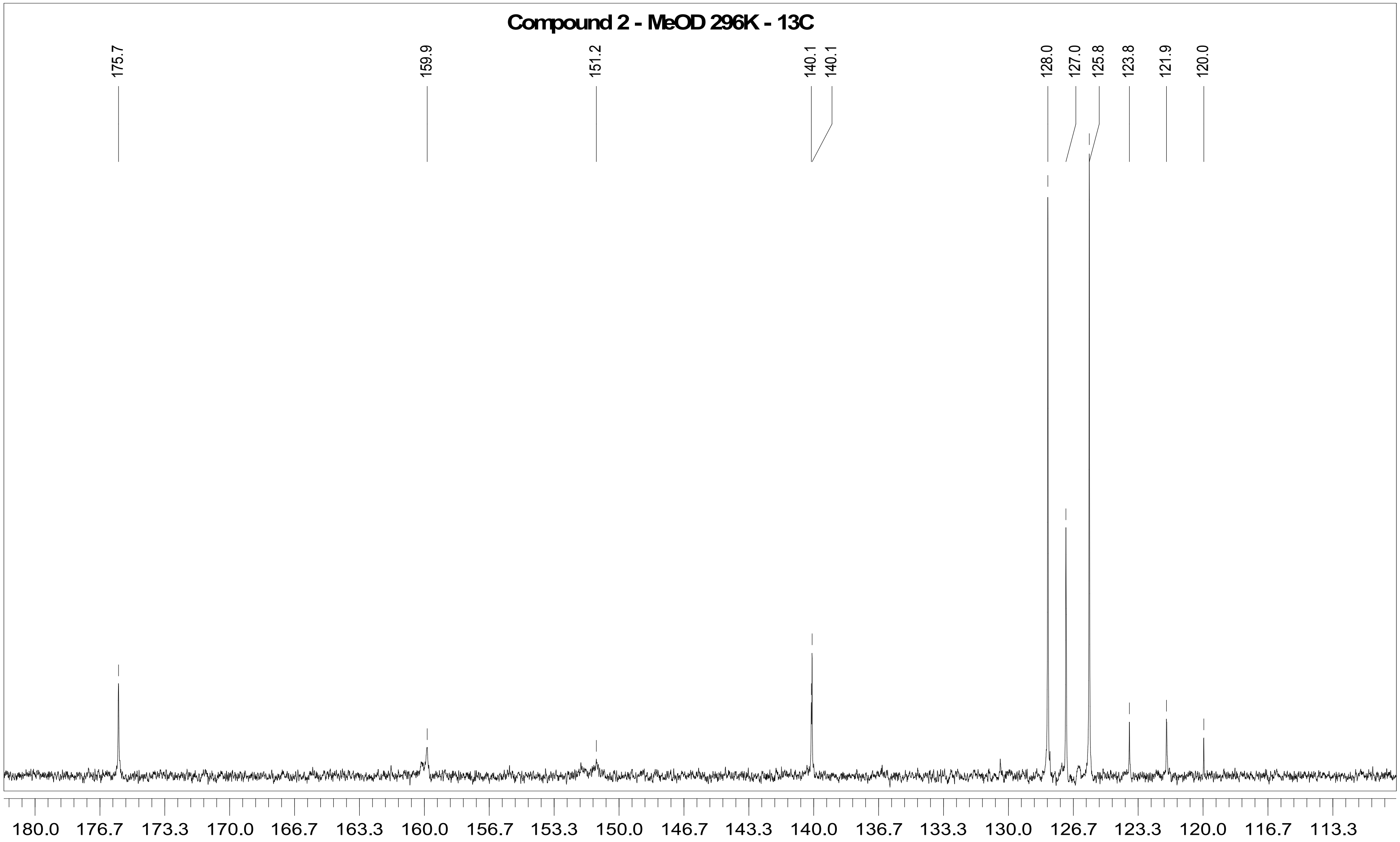
Compound 2 - MeOD 296K - 13C
175.7
159.9
151.2
140.1
140.1
128.0
127.0
125.8
123.8
121.9
120.0
180.0 176.7 173.3 170.0 166.7 163.3 160.0 156.7 153.3 150.0 146.7 143.3 140.0 136.7 133.3 130.0 126.7 123.3 120.0 116.7 113.3

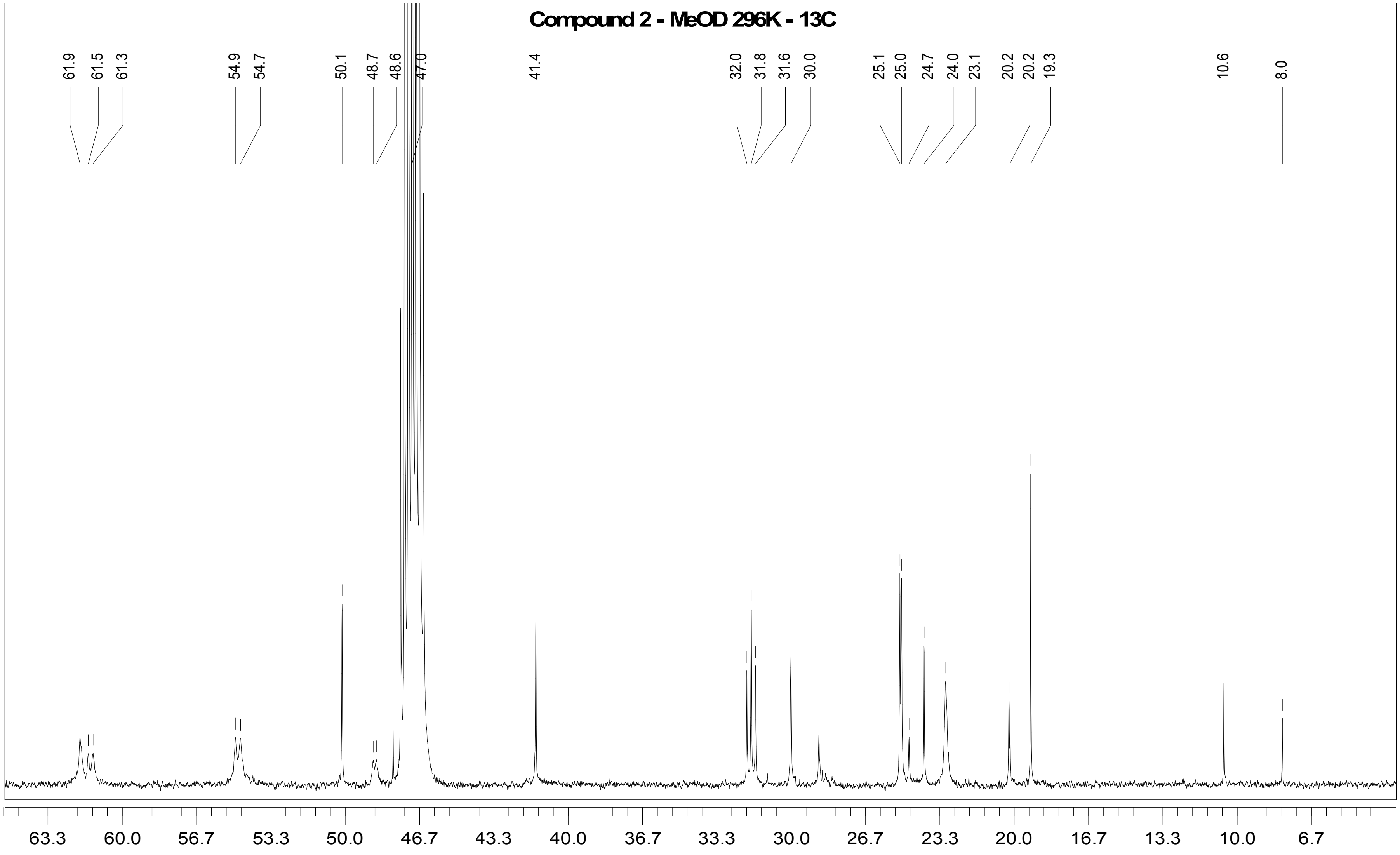
Compound 2 - MeOD 296K - 13C
61.9
61.5
61.3
54.9
54.7
50.1
48.7
48.6
47.0
41.4
32.0
31.8
31.6
30.0
25.1
25.0
24.7
24.0
23.1
20.2
20.2
19.3
10.6
8.0
63.3
60.0
56.7
53.3
50.0
46.7
43.3
40.0
36.7
33.3
30.0
26.7
23.3
20.0
16.7
13.3
10.0
6.7

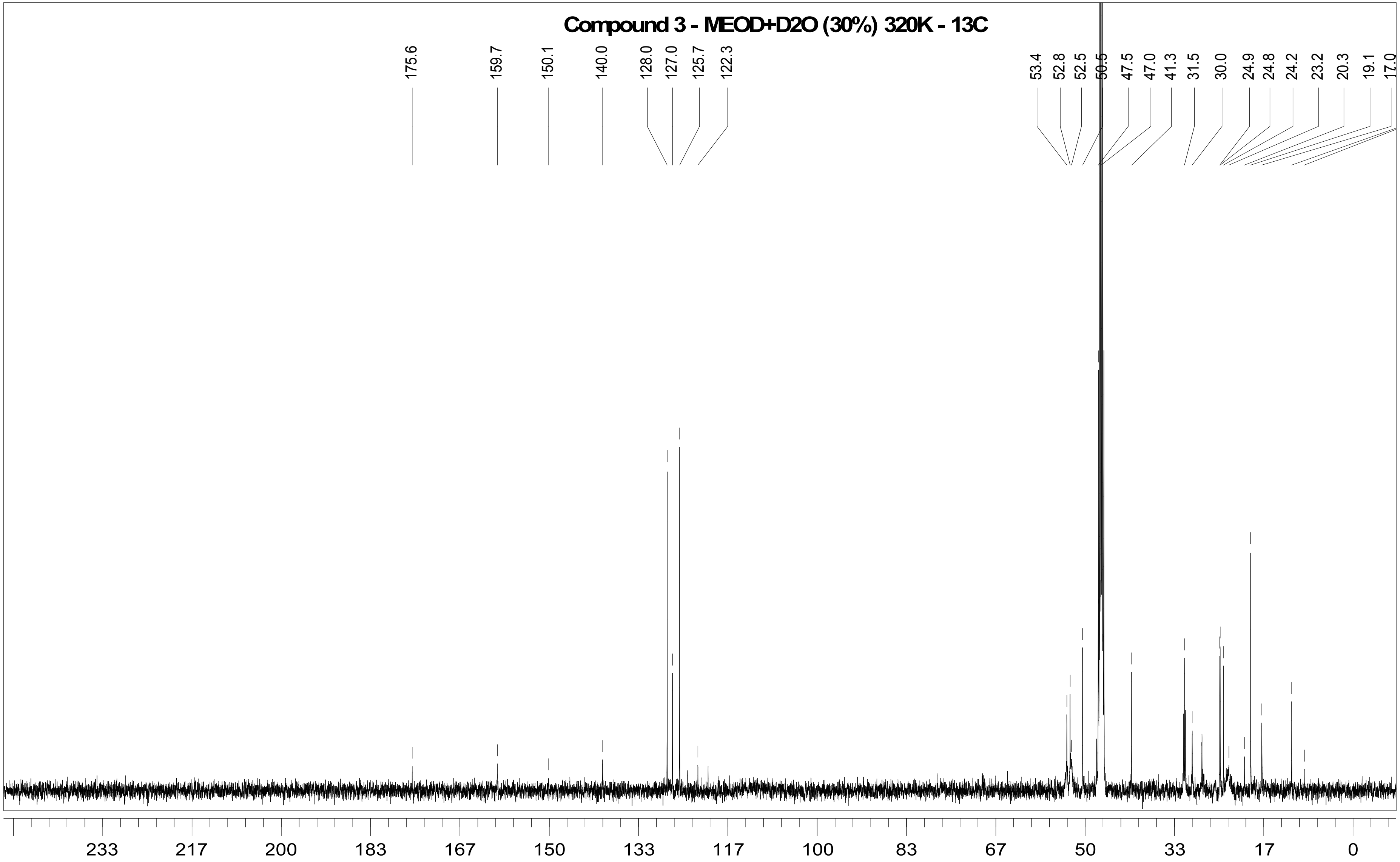

Compound 3 - MEOD+D2O (30%) 320K - 13C
175.6
159.7
150.1
140.0
128.0
127.0
125.7
122.3
53.4
52.8
52.5
50.5
47.5
47.0
41.3
31.5
30.0
24.9
24.8
24.2
23.2
20.3
19.1
17.0
233
217
200
183
167
150
133
117
100
83
67
50
33
17
0

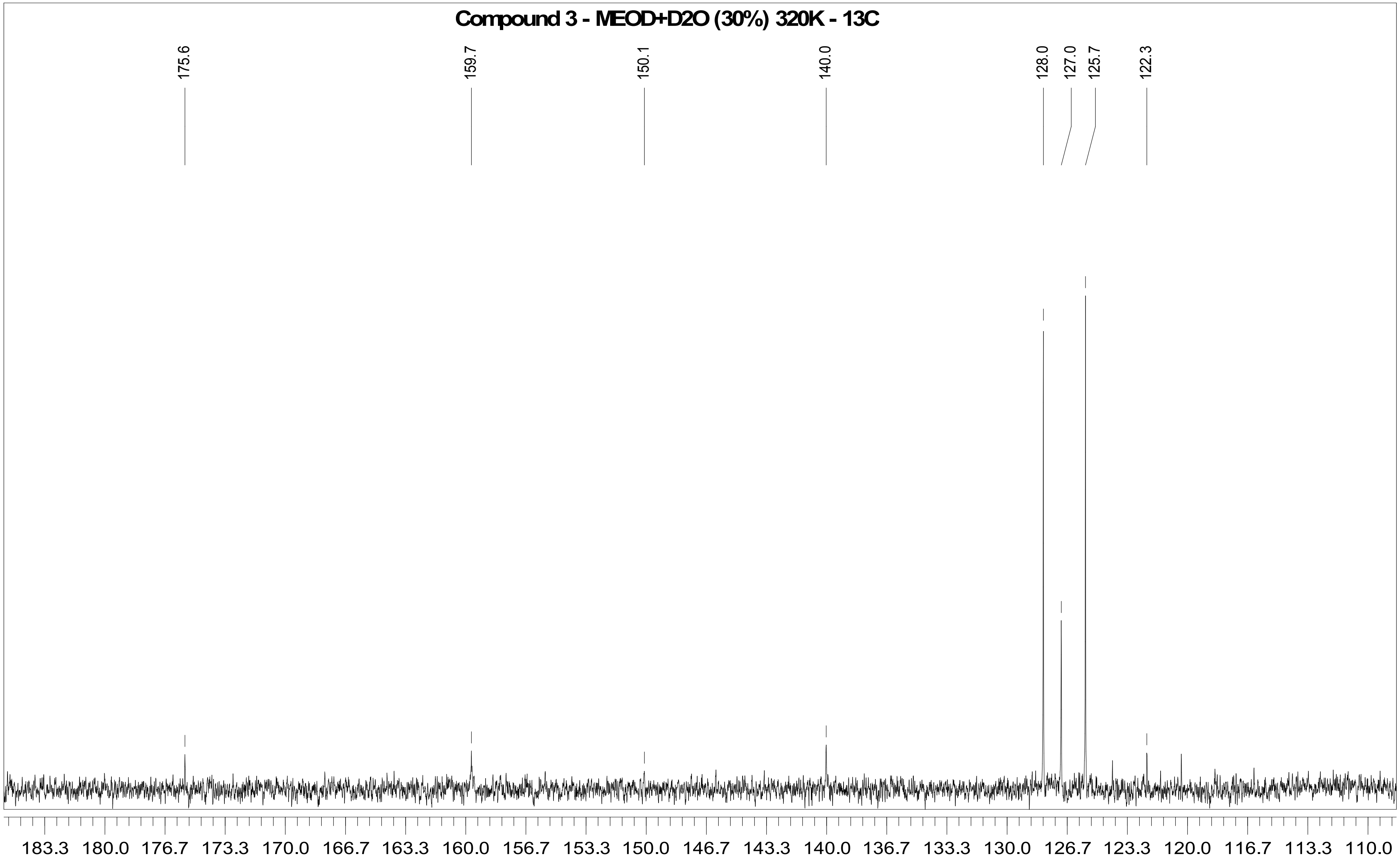

Compound 3 - MEOD+D2O (30%) 320K - 13C
175.6
159.7
150.1
140.0
128.0
127.0
125.7
122.3
183.3 180.0 176.7 173.3 170.0 166.7 163.3 160.0 156.7 153.3 150.0 146.7 143.3 140.0 136.7 133.3 130.0 126.7 123.3 120.0 116.7 113.3 110.0

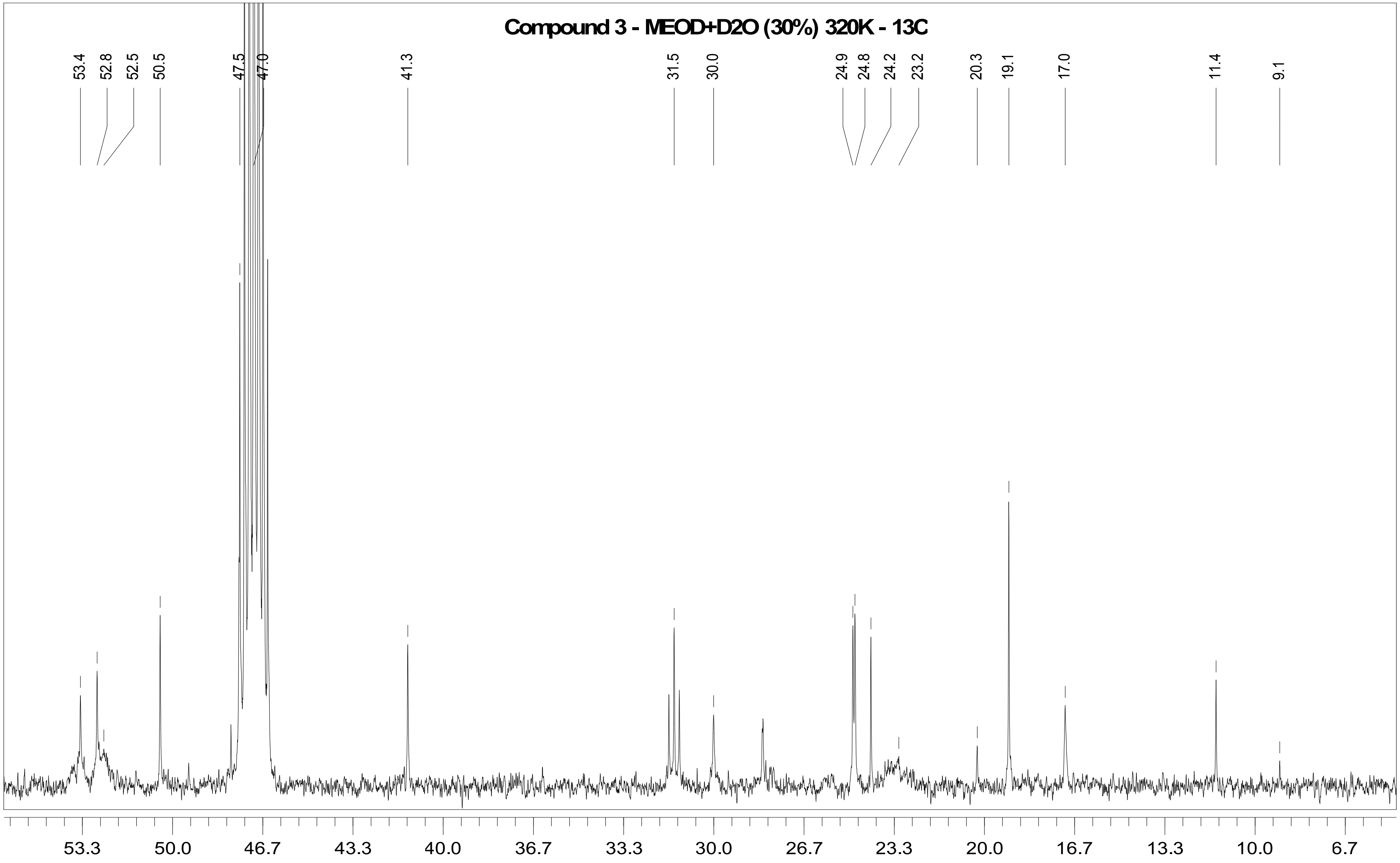
Compound 3 - MEOD+D2O (30%) 320K - 13C
53.4
52.8
52.5
50.5
47.5
47.0
41.3
31.5
30.0
24.9
24.8
24.2
23.2
20.3
19.1
17.0
11.4
9.1
53.3
50.0
46.7
43.3
40.0
36.7
33.3
30.0
26.7
23.3
20.0
16.7
13.3
10.0
6.7

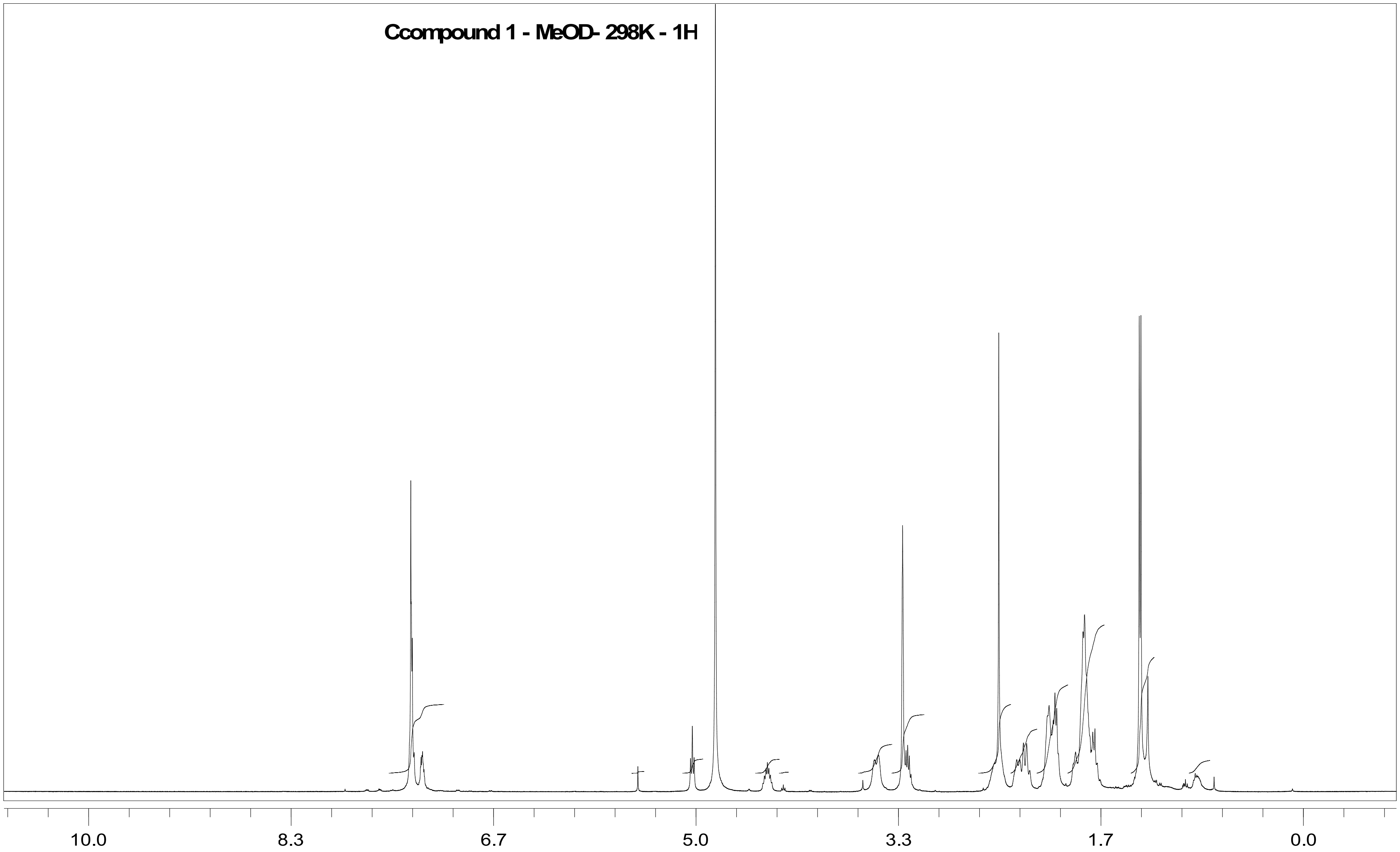

Ccompound 1 - MeOD- 298K - 1H
10.0
8.3
6.7
5.0
3.3
1.7
0.0

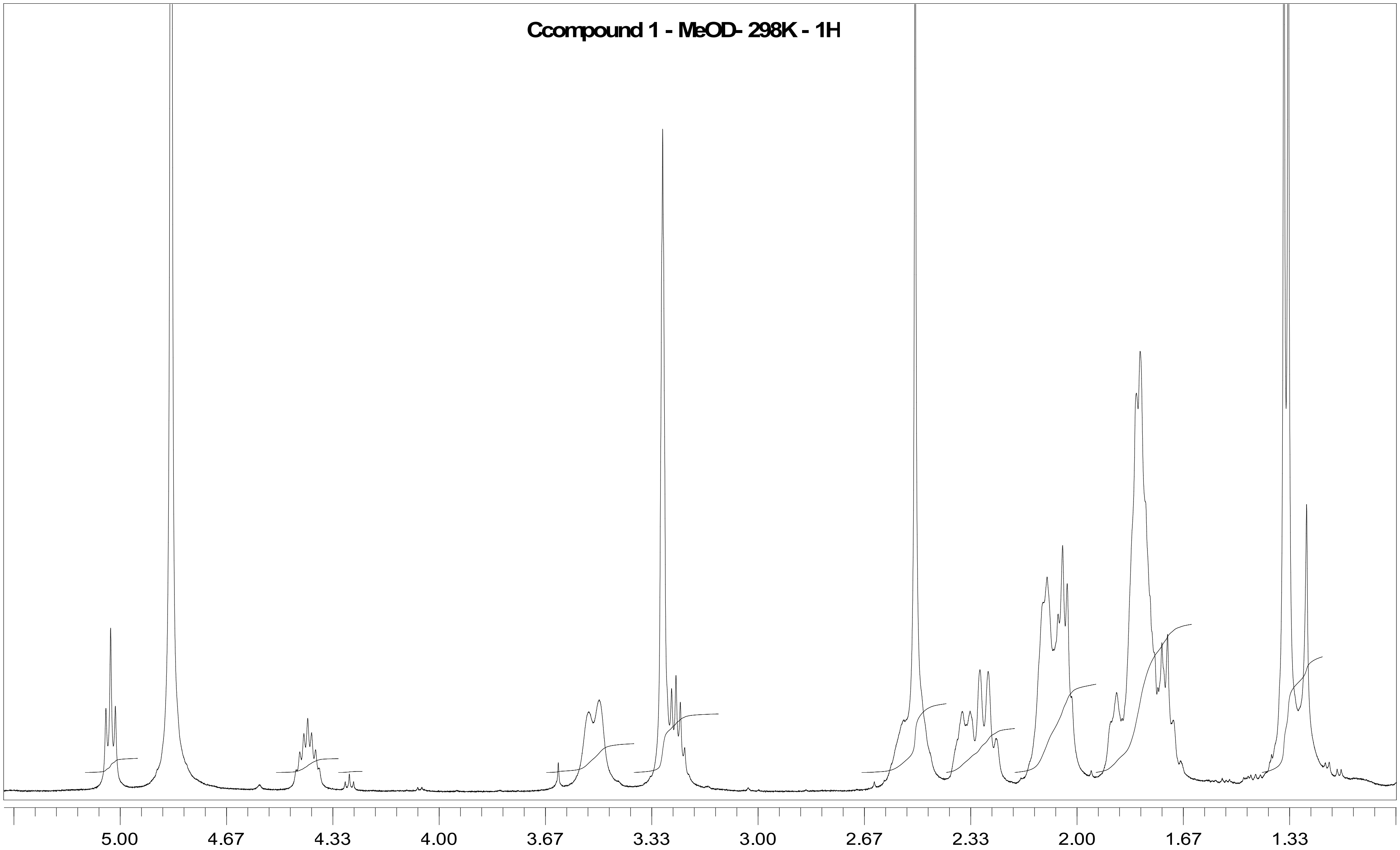
Ccompound 1 - MeOD- 298K - 1H
5.00
4.67
4.33
4.00
3.67
3.33
3.00
2.67
2.33
2.00
1.67
1.33

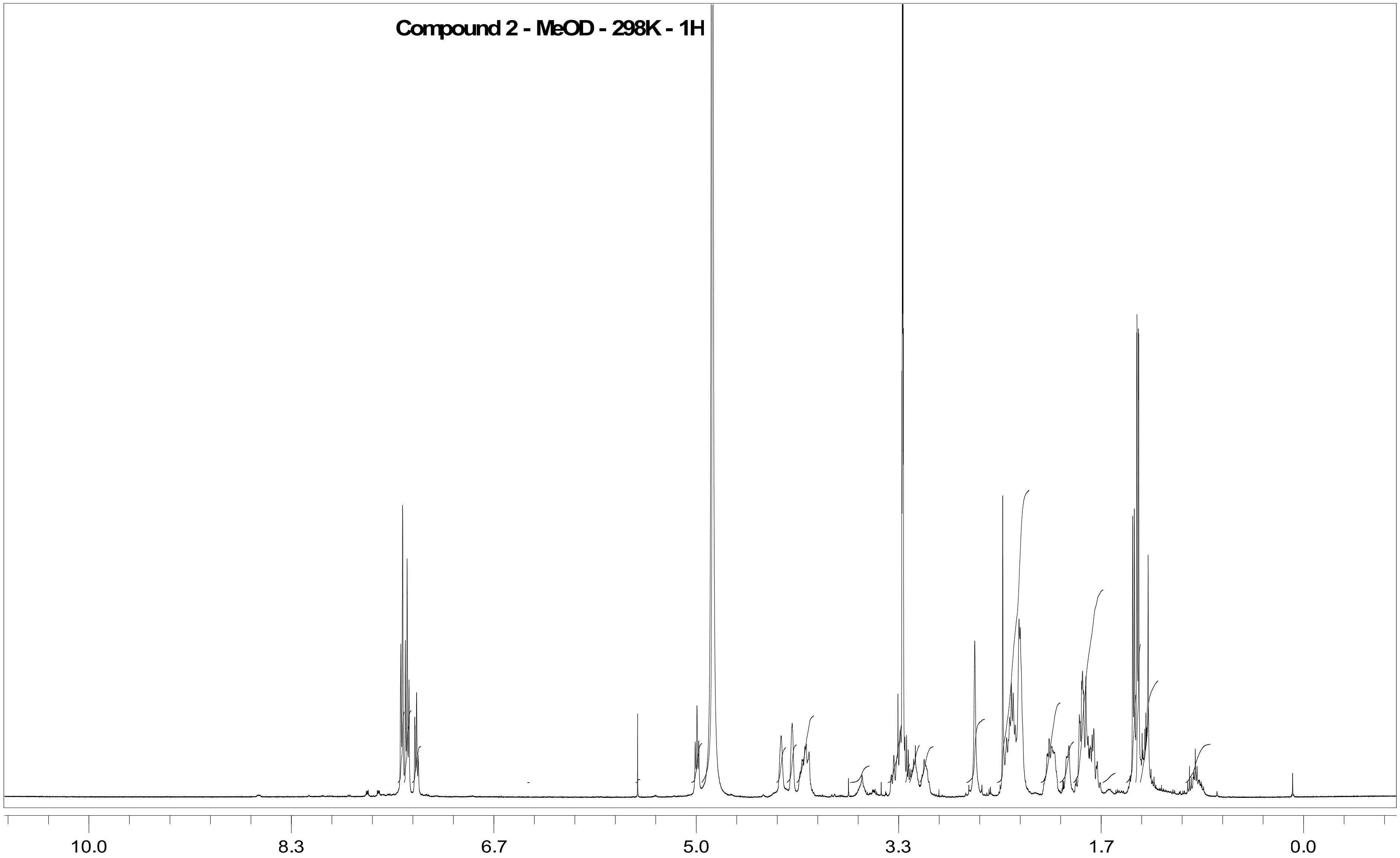
Compound 2 - MeOD - 298K - 1H
10.0
8.3
6.7
5.0
3.3
1.7
0.0

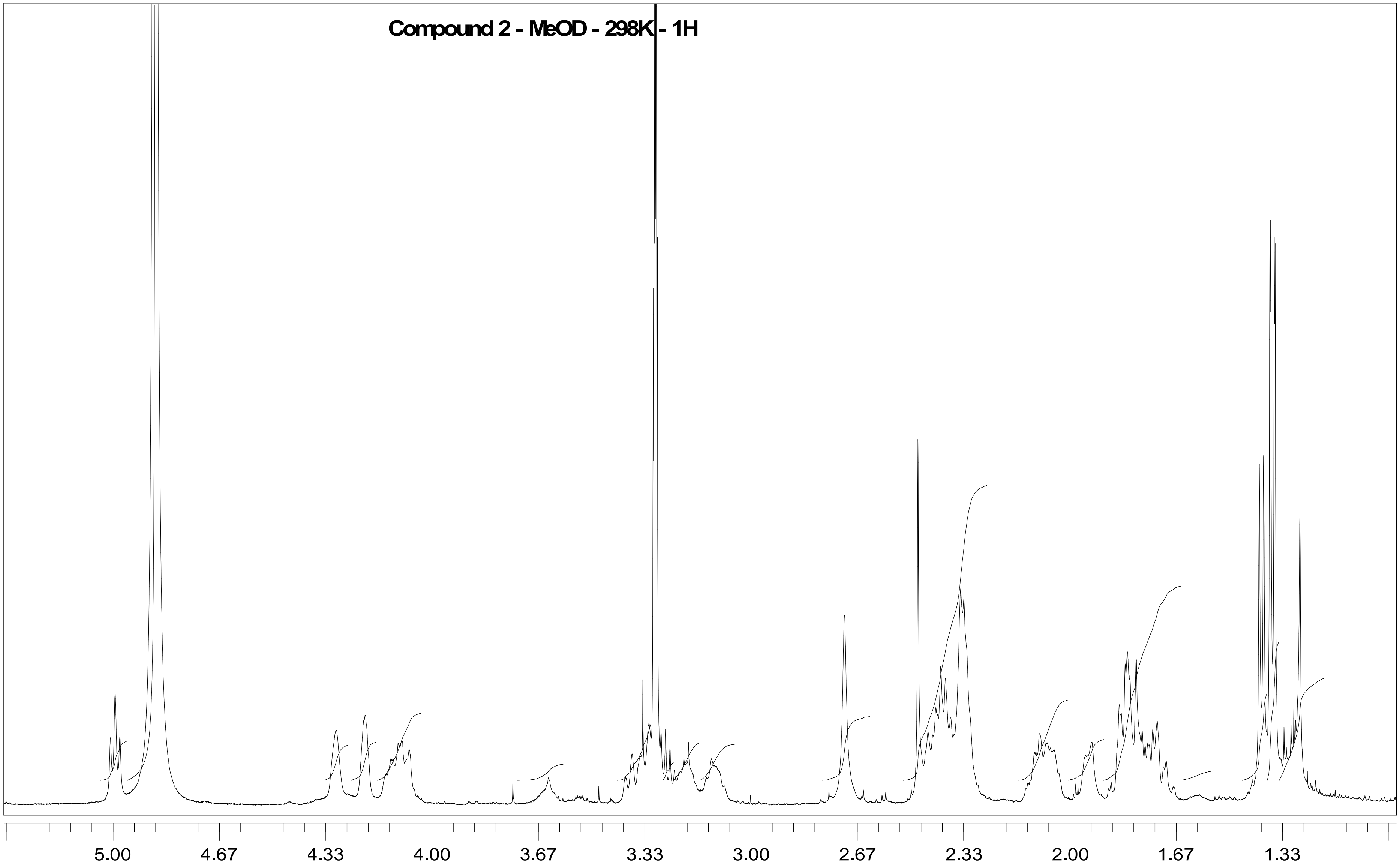
Compound 2 - MeOD - 298K - 1H
5.00
4.67
4.33
4.00
3.67
3.33
3.00
2.67
2.33
2.00
1.67
1.33

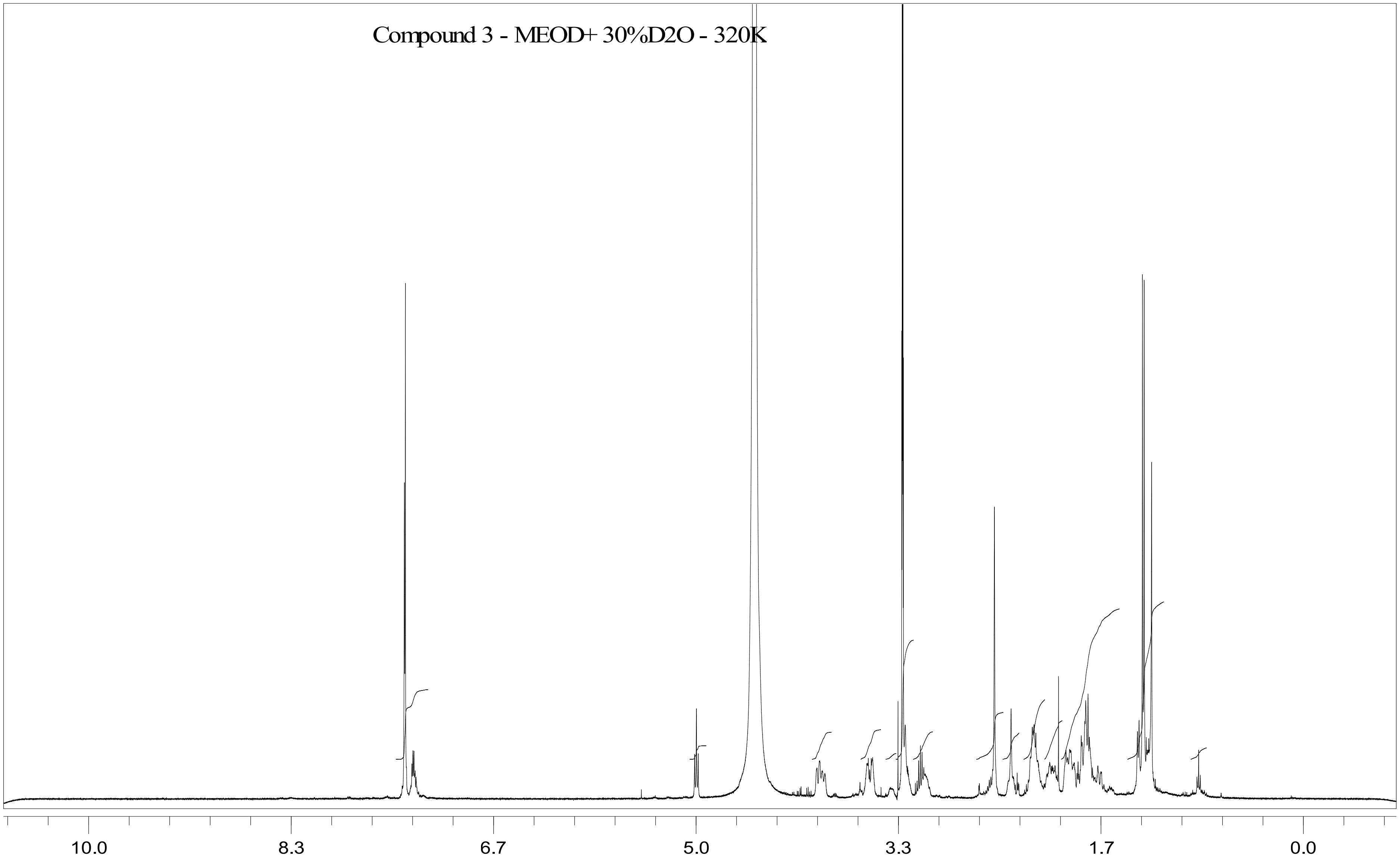
Compound 3 - MEOD+ 30%D2O - 320K
10.0
8.3
6.7
5.0
3.3
1.7
0.0

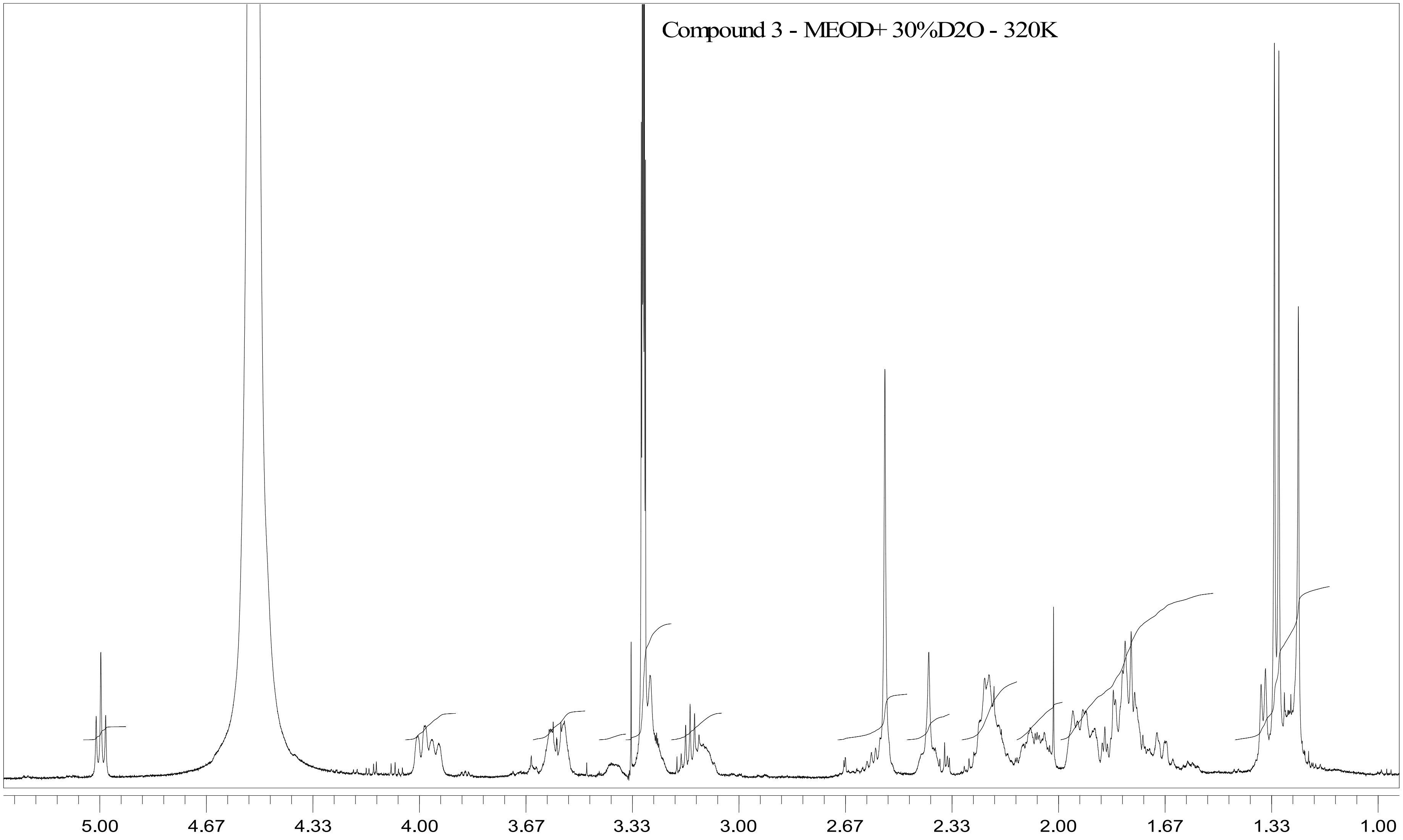

Compound 3 - MEOD+ 30%D2O - 320K
5.00
4.67
4.33
4.00
3.67
3.33
3.00
2.67
2.33
2.00
1.67
1.33
1.00

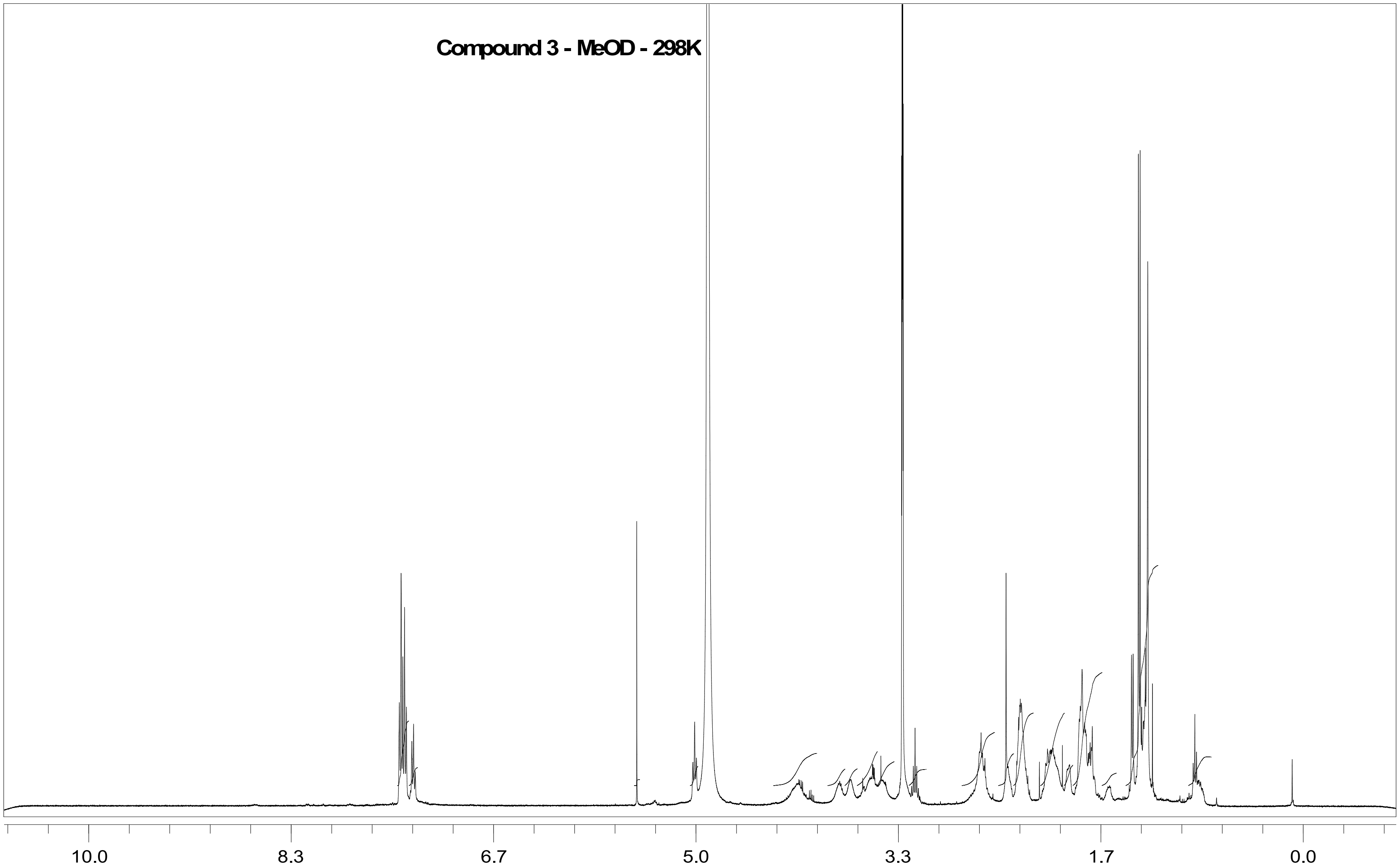
Compound 3 - MeOD - 298K
10.0
8.3
6.7
5.0
3.3
1.7
0.0

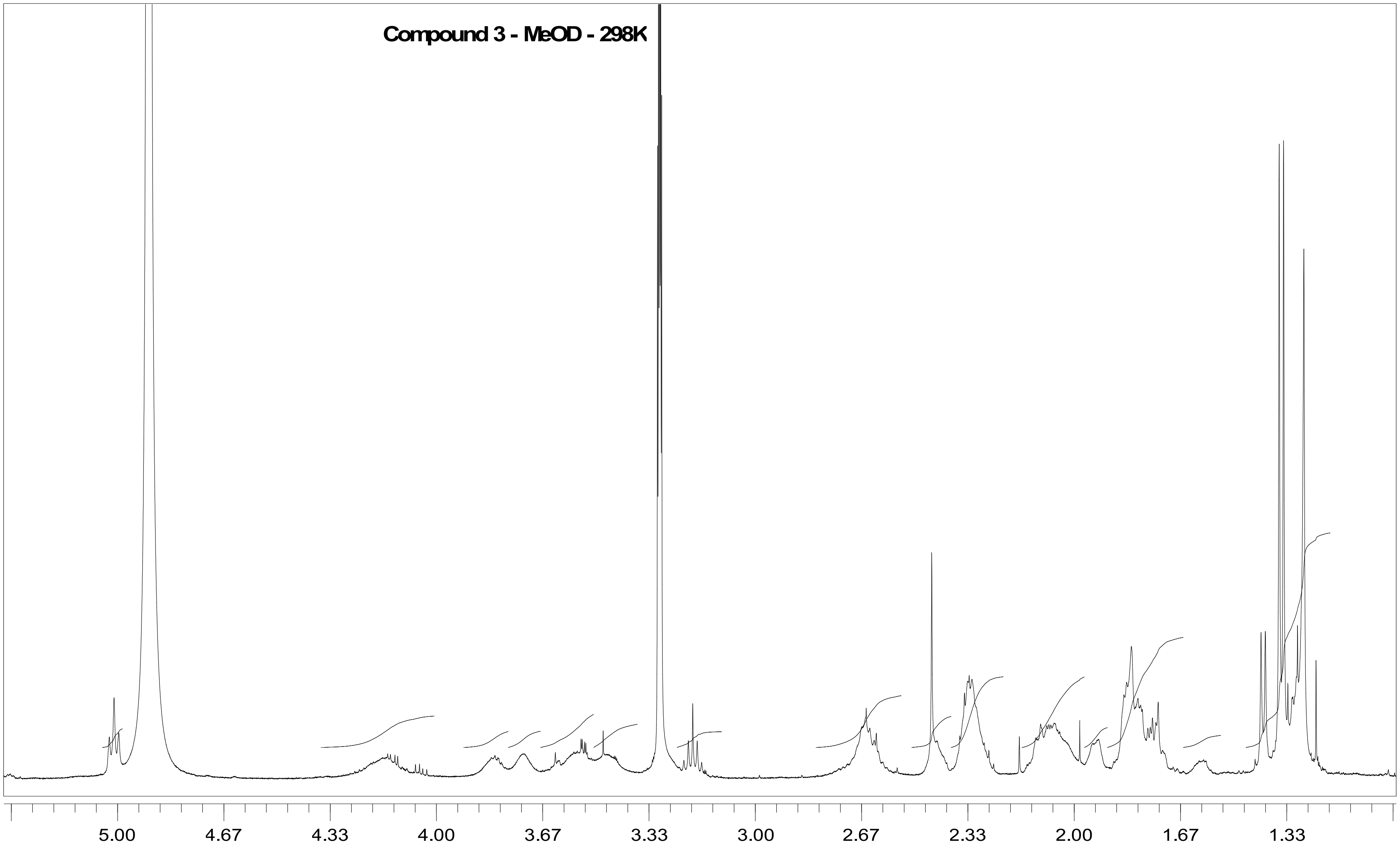
Compound 3 - MeOD - 298K
5.00
4.67
4.33
4.00
3.67
3.33
3.00
2.67
2.33
2.00
1.67
1.33

Assignments of the axial and equatorial protons of 1-3

Equatorial H-2/H-4 of compound 1 were given at 1.81 ppm due to their NOESY cross peaks with H-3 (4.41 ppm); consequently the axial H-2/H-4 were assigned at 2.29 ppm. Similar correlations were observed between equatorial H-2/H-4 at 3.32 and 3.35 ppm and H-6/H-7 at 2.34 of compound 2 and between equatorial H-2/H-4 at 3.29 and equatorial H-6/H-8 at 1.95 ppm of compound 3.

Table S1. Relative energies (kcal/mol), equilibrium percentages at 298 K, and significant torsional angles[a] (°) of significantly populated conformers (P_i > 1%) of compound **1**.

	E_{rel} (kcal/mol)	P_i(%)	τ_1 (°)[a]	τ_2 (°)[b]	τ_3 (°)[c]	τ_4 (°)[d]	τ_5 (°)[e]	τ_6 (°)[e]	τ_7 (°)[g]	τ_8 (°)[h]
**1A**	0.00	39.3	-4	-164	67	165	-60	-168	-112	41
**1B**	0.12	32.3	-4	-165	67	165	-60	-168	-125	-41
**1C**	0.92	8.4	-3	-164	68	168	-60	-167	64	2
**1D**	1.13	5.8	-2	-162	77	105	-63	-65	63	7
**1E**	1.38	3.8	-1	-162	77	103	-64	-65	-112	41
**1F**	1.52	3.0	-2	-162	77	103	-64	-66	-127	-41
**1G**	1.67	2.3	37	-162	67	174	-61	-76	-123	-41
**1H**	1.81	1.8	24	-162	67	173	-61	-76	-108	40

[a] τ_1: H-C1”-C1’’a-N; τ_2: C1’’a-N-C8c-C1’; τ_3: C1’-C8c-C8b-C8a; τ_4: C8c-C8b-C8a-N8; τ_5: N-C8c-C1’- C2’; τ_6: C8b-C8a-N8-C5; τ_7: C4-C3-N1’’’-C5’’’ ; τ_8: N1’’’-C5’’’-C5’’’a-H.

Figure S1. 3D-plots of conformers **C-H** of compound **1**.

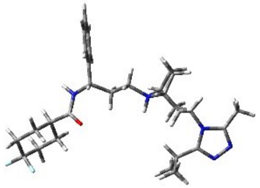

1C

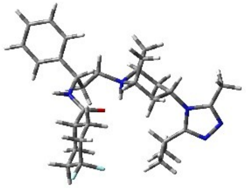

1D

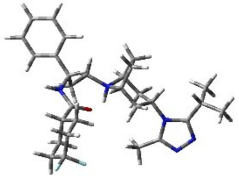

1E

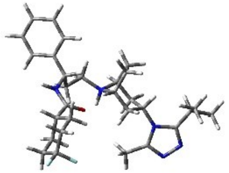

1F

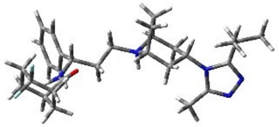

1G

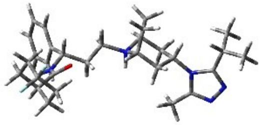

1H

Table S2. Relative energies (kcal/mol), equilibrium percentages at 298 K, and torsional angles[a] (°) of significantly populated conformers (P_i > 1%) of compounds **2** and **3**.

	E_{rel} (kcal/mol)	P_i(%)	τ_1 (°)[a]	τ_2 (°)[b]	τ_3 (°)[c]	τ_4 (°)[d]	τ_5 (°)[e]	τ_6 (°)[f]	τ_7 (°)[g]	τ_8 (°)[h]
**2A**	0.37	21.1	7	-163	66	169	-60	-169	-111	29
**2B**	0.20	28.0	7	-163	67	168	-60	-169	-114	-31
**2C**	0.00	39.3	-2	-164	68	165	-60	-168	68	11
**2D**	1.47	3.3	-2	-162	77	103	-63	-66	68	9
**2E**	1.79	1.9	-3	-162	78	102	-63	-67	-112	31
**2F**	1.68	2.3	0	-162	78	101	-63	-67	-115	-31
**3A**	0.88	7.0	7	-163	69	168	-60	-161	-110	37
**3B**	0.62	11.0	7	-163	65	165	-60	-166	-118	-36
**3C**	0.00	31.2	-1	-164	66	160	-61	-167	67	-11
**3D**	0.10	26.1	7	-163	67	175	-60	-71	67	-3
**3E**	0.73	9.1	8	-163	67	178	-61	-69	-110	36
**3F**	0.76	8.7	9	-163	69	-179	-60	-67	-118	-36
**3I**	1.51	2.4	4	-163	66	174	-60	-167	67	8

[a] τ1: H-C1”-C1’’a-N; τ2: C1’’a-N-C8c-C1’ for **2**, C1’’a-N-C9c-C1’ for **3**; τ3: C1’-C8c-C8b-C8a for **2**, C1’-C9c-C9b-C9a for **3**; τ4: C8c-C8b-C8a-N8 for **2**, C9c-C9b-C9a-N9 for **3**; τ5: N-C8c-C1’- C2’ for **2**, τ5: N-C9c-C1’- C2’ for **3**; τ6: C8b-C8a-N8-C5 for **2**, C9b-C9a-N9-C5 for **3**; τ7: C4-C3-N1’”-C5’”; τ8: N1’”-C5’”-C5’’’a-H.

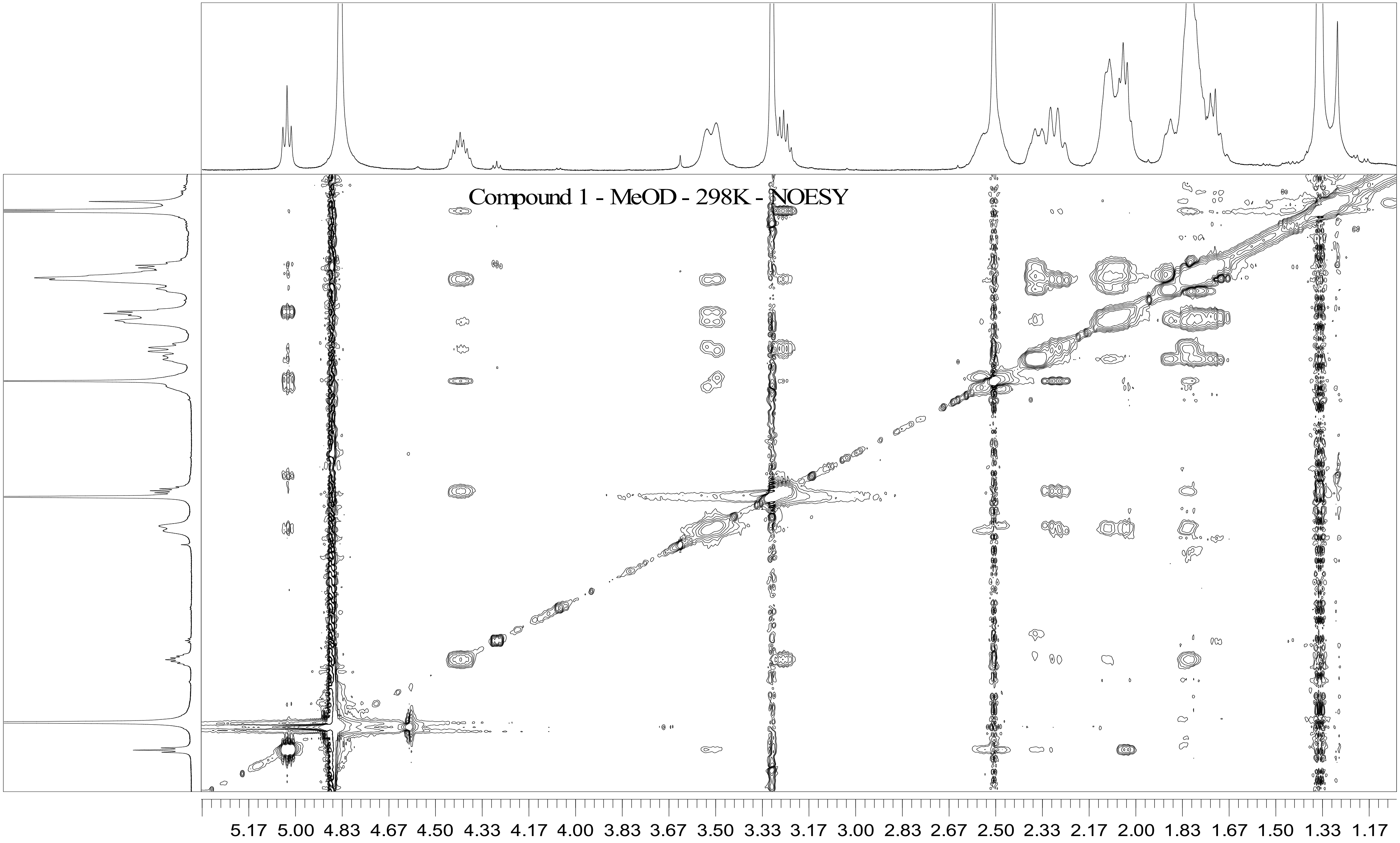
Compound 1 - MeOD - 298K - NOESY
5.17 5.00 4.83 4.67 4.50 4.33 4.17 4.00 3.83 3.67 3.50 3.33 3.17 3.00 2.83 2.67 2.50 2.33 2.17 2.00 1.83 1.67 1.50 1.33 1.17

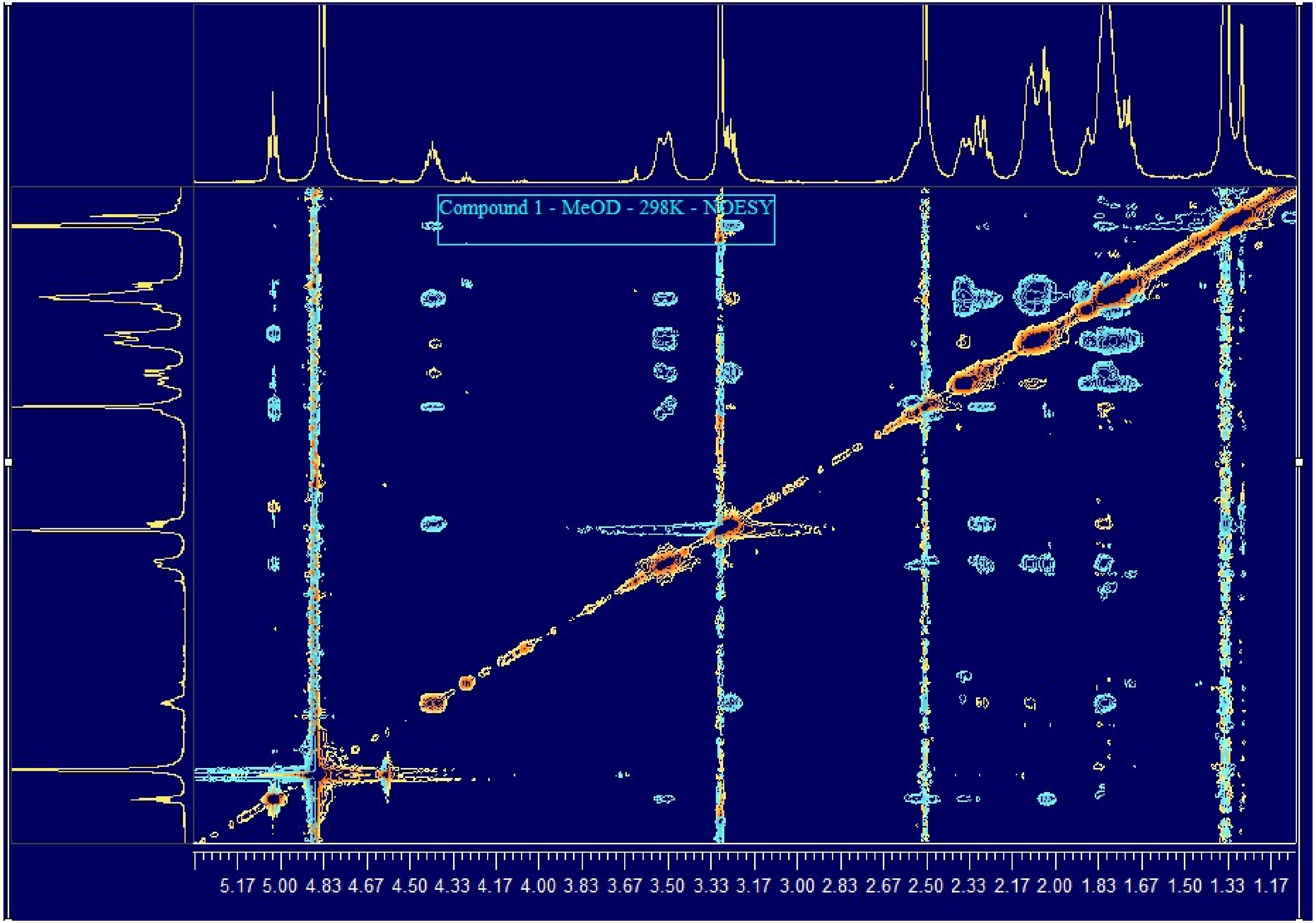
Compound 1 - MeOD - 298K - NOESY
5.17 5.00 4.83 4.67 4.50 4.33 4.17 4.00 3.83 3.67 3.50 3.33 3.17 3.00 2.83 2.67 2.50 2.33 2.17 2.00 1.83 1.67 1.50 1.33 1.17

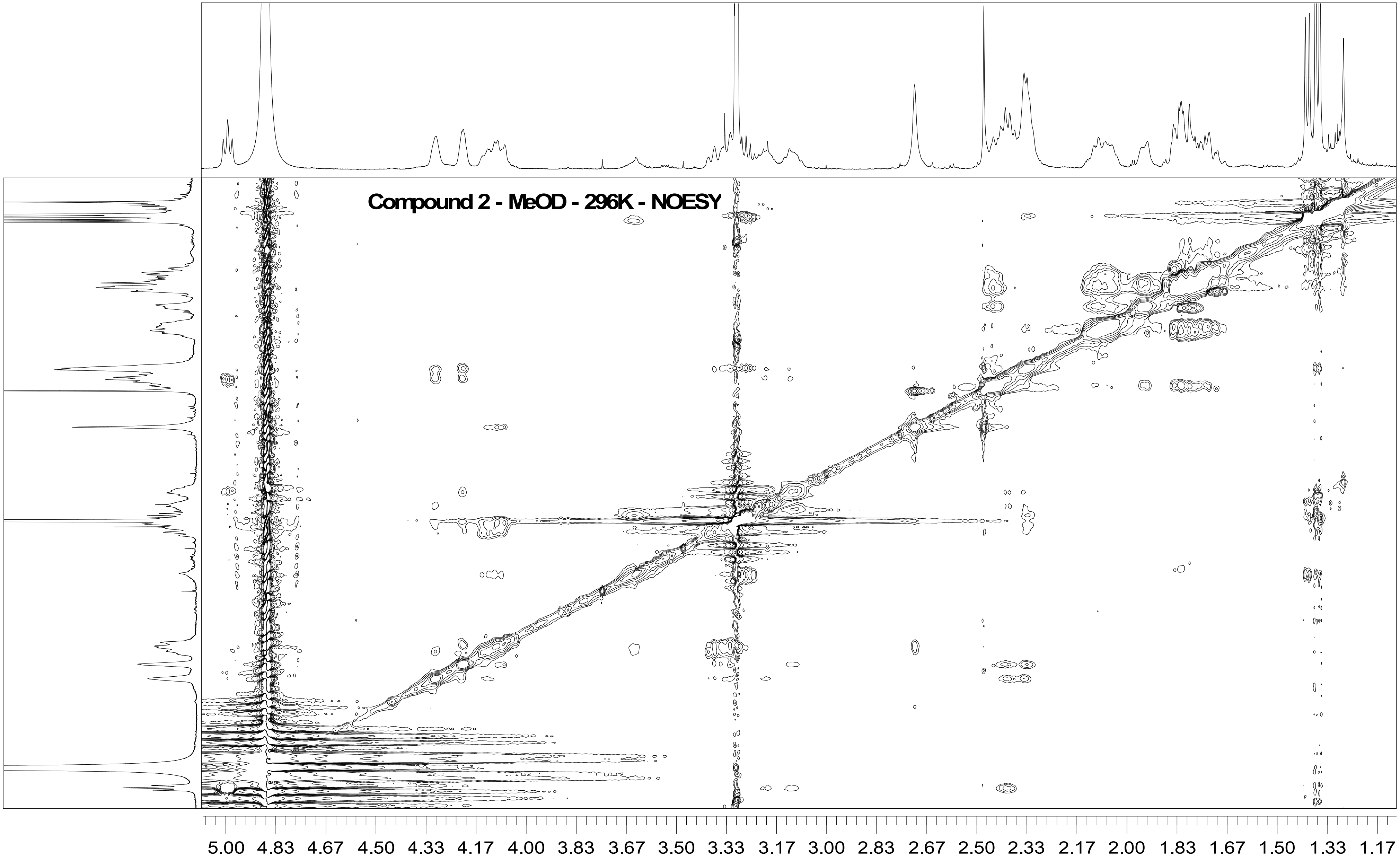
Compound 2 - MeOD - 296K - NOESY
5.00 4.83 4.67 4.50 4.33 4.17 4.00 3.83 3.67 3.50 3.33 3.17 3.00 2.83 2.67 2.50 2.33 2.17 2.00 1.83 1.67 1.50 1.33 1.17

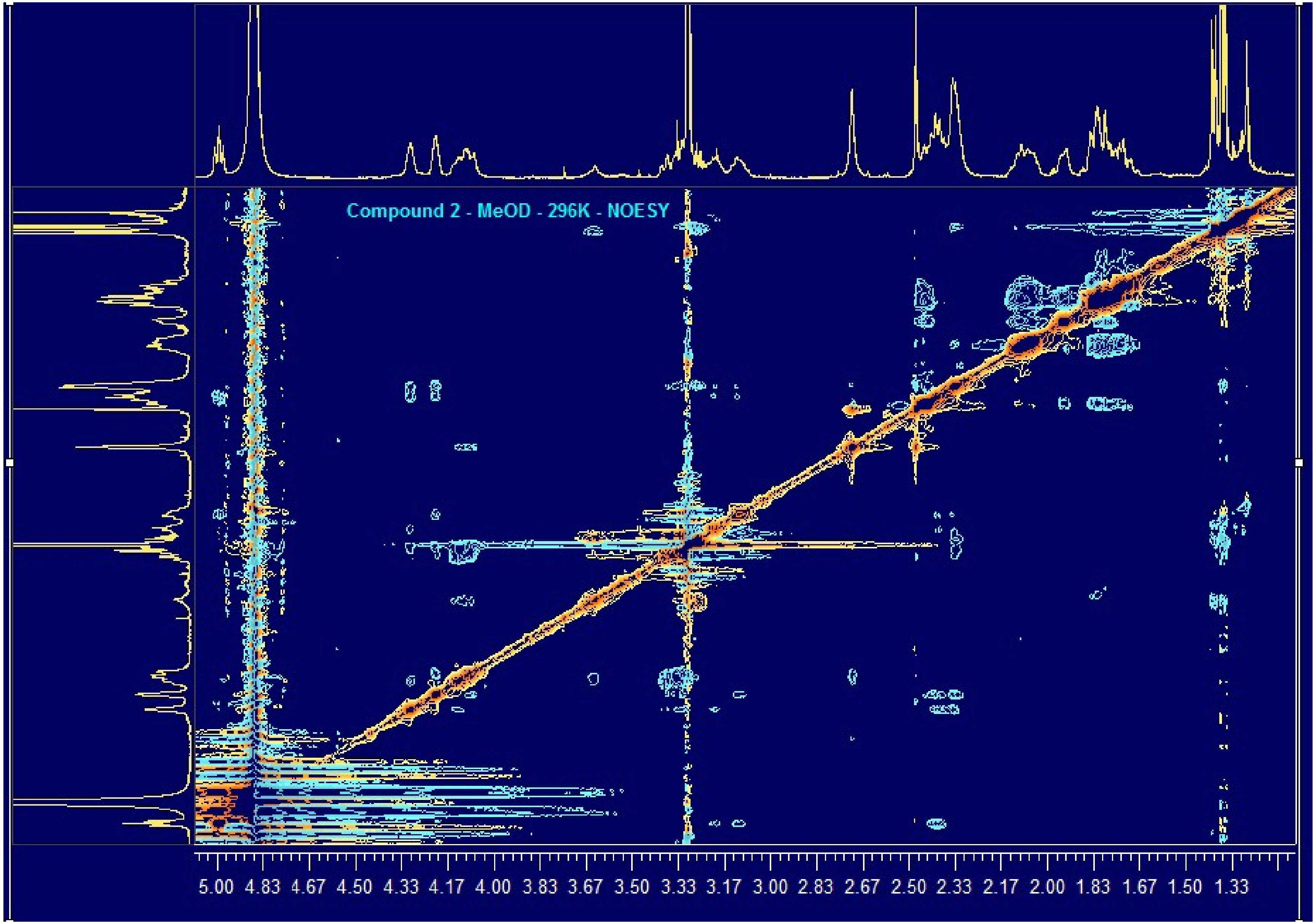
Compound 2 - MeOD - 296K - NOESY
5.00 4.83 4.67 4.50 4.33 4.17 4.00 3.83 3.67 3.50 3.33 3.17 3.00 2.83 2.67 2.50 2.33 2.17 2.00 1.83 1.67 1.50 1.33

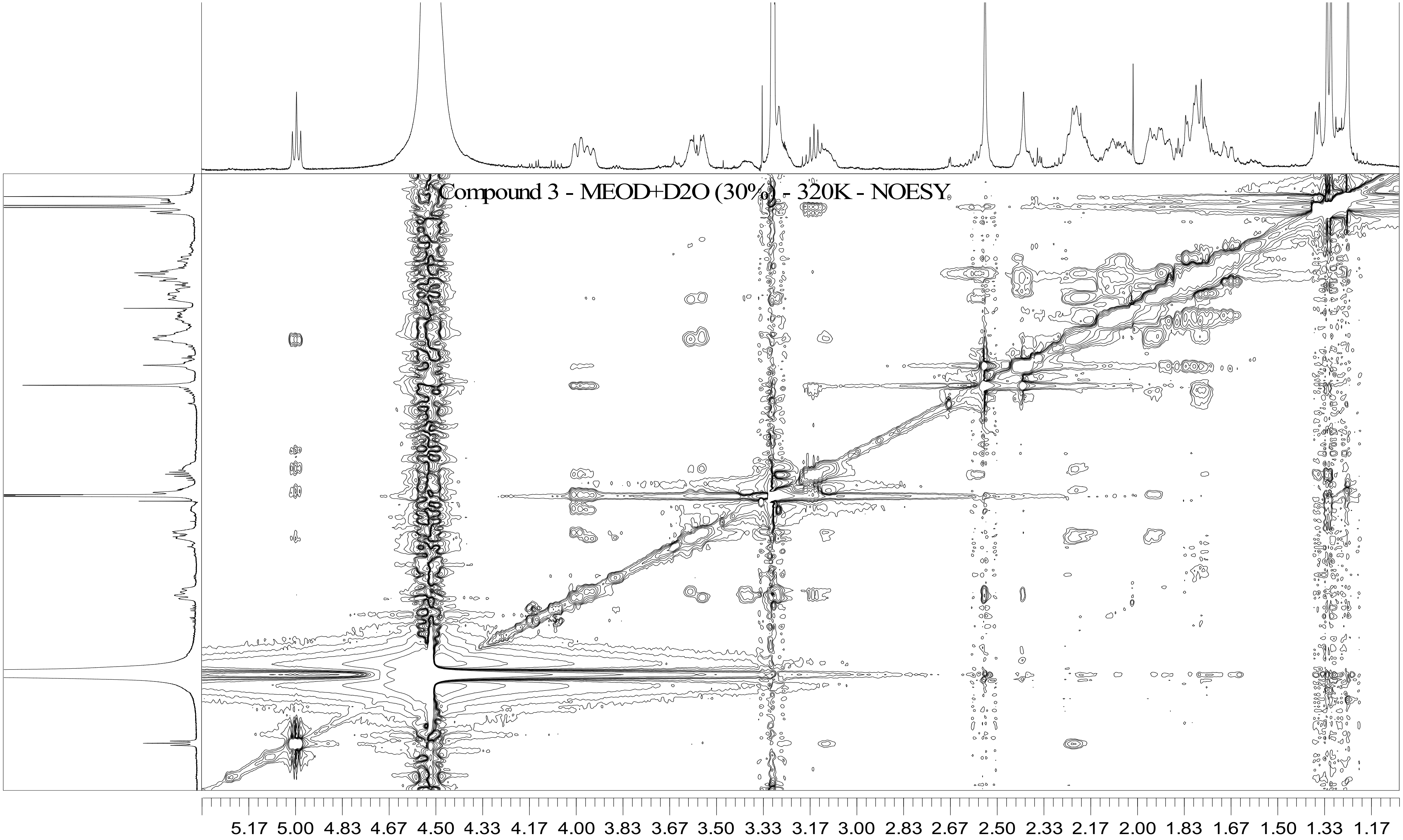
Compound 3 - MEOD+D2O (30%) - 320K - NOESY
5.17 5.00 4.83 4.67 4.50 4.33 4.17 4.00 3.83 3.67 3.50 3.33 3.17 3.00 2.83 2.67 2.50 2.33 2.17 2.00 1.83 1.67 1.50 1.33 1.17

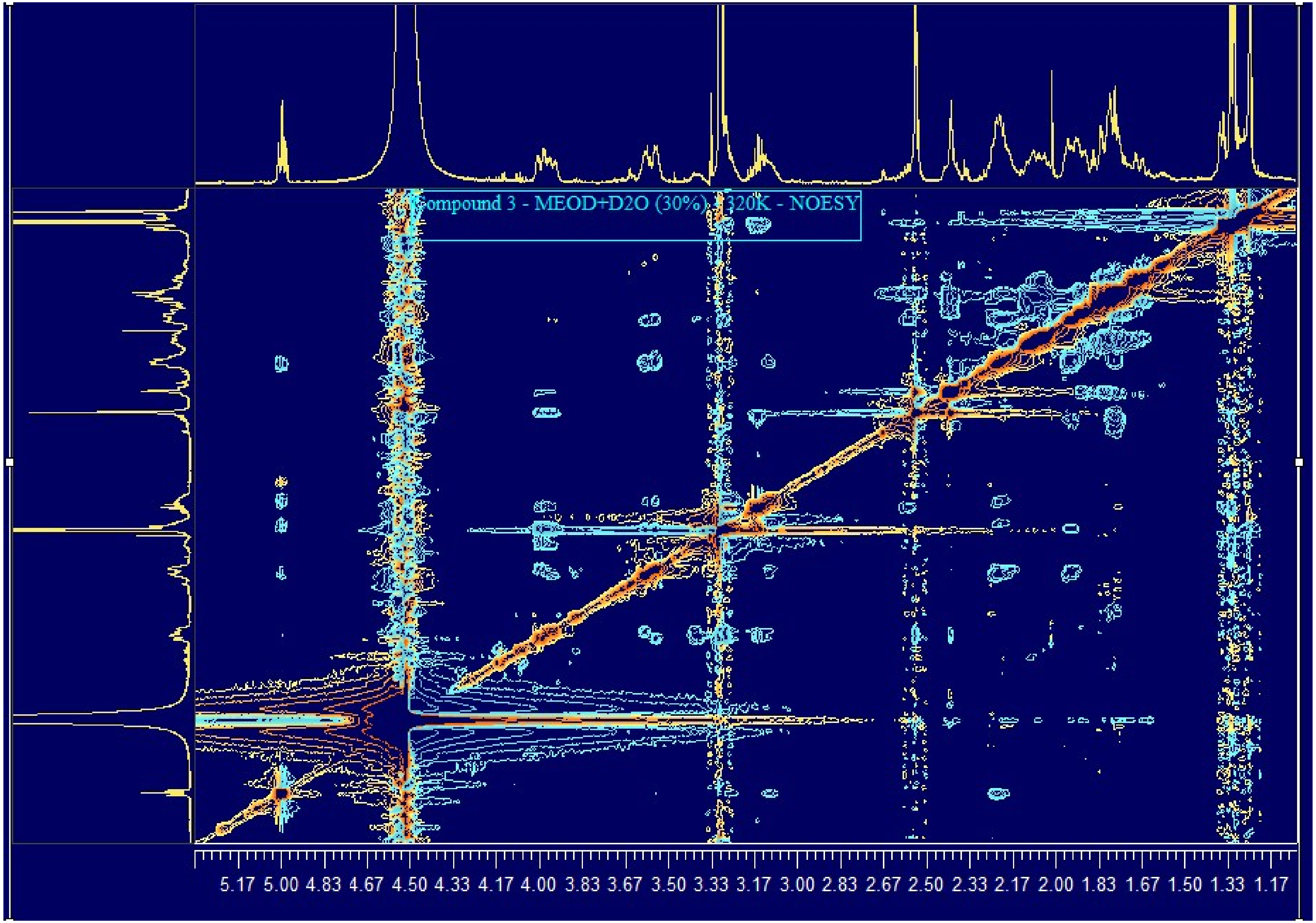
Compound 3 - MEOD+D2O (30%) - 320K - NOESY
5.17 5.00 4.83 4.67 4.50 4.33 4.17 4.00 3.83 3.67 3.50 3.33 3.17 3.00 2.83 2.67 2.50 2.33 2.17 2.00 1.83 1.67 1.50 1.33 1.17

^{1}H-NMR spectra of **2** recorded at increasing temperatures

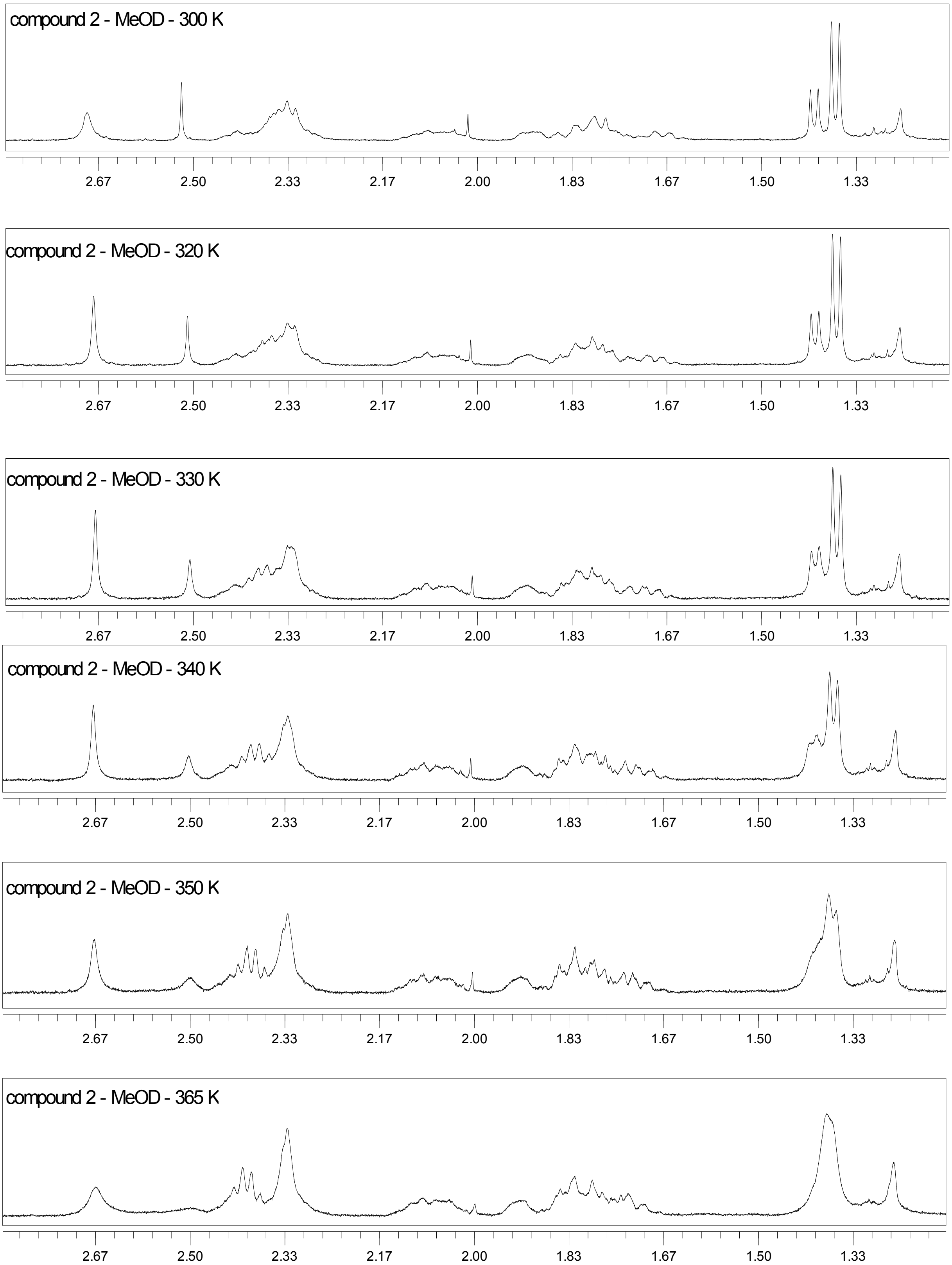

Table S3. ^{1}H NMR chemical shift (δ) of compounds **1**-**3**.

^{1}H position	δ(ppm)		
	(**1**) [a] Exp	(**2**) [a] Exp	(**3**) [b] Exp
1 (5)	3.50	4.30	3.59
2-ax (4-ax)	2.29	4.11	3.99
2-eq (4-eq)	1.81	3.32	3.29
3	4.41	-	-
4-ax (2-ax)	2.29	4.11	3.95
4-eq (2-eq)	1.81	3.35	3.29
5 (1)	3.53	4.21	3.55
6-ax	2.10	2.34	2.20
6-eq	2.10	2.34	1.95
7-ax	2.10	2.34	2.58
7-eq	2.10	2.34	1.79
8-ax	-	-	2.20
8-eq	-	-	1.95
8a (9a)	2.54	3.11	3.11
8a (9a)	2.54	3.19	3.26
8b (9b)	2.03	2.35	2.24
8b (9b)	2.03	2.42	2.24
8c (9c)	5.03	4.99	4.99
1”	2.35	2.45	2.40
2”,3”,5”,6”	1.68-2.18	1.65-2.15	1.64-2.13
2’-6’	7.23-7.34	7.27-7.45	7.27-7.43
2CH_3(*i*Pr)	1.35	1.36	1.32
2CH_3(*i*Pr)	-	1.40	1.36
CH_3	2.51	2.70	2.54
CH_3	-	2.47	2.40
5’’’a	3.26	3.26	3.15
5’’’a	-	3.63	3.29

(a) CD_3OD, 298 K

(b) CD_3OD:D_2O 70:30, 320 K
